# Experimental and Computational Study of Injectable Iron(III)/Ultrashort Peptide Hydrogels: A Candidate for Ferroptosis‐Induced Treatment of Bacterial Infections

**DOI:** 10.1002/smsc.202400618

**Published:** 2025-04-17

**Authors:** Capucine Loth, Florent Barbault, Cécile Guégan, Flora Lemaire, Christophe Contal, Alain Carvalho, Sophie Hellé, Marie Champion, Halima Kerdjoudj, Delphine Chan‐Seng, Lydie Ploux, Fouzia Boulmedais

**Affiliations:** ^1^ Université de Strasbourg CNRS Institut Charles Sadron UPR 22 F‐67000 Strasbourg France; ^2^ Université Paris Cité CNRS ITODYS F‐75013 Paris France; ^3^ Inserm UMR_S 1121 CNRS EMR 7003 Biomaterials and Bioengineering Université de Strasbourg Centre de Recherche en Biomédecine de Strasbourg 1 rue Eugène Boeckel F‐67000 Strasbourg France; ^4^ Biomatériaux et Inflammation en Site Osseux (BIOS) EA 4691 Université de Reims Champagne Ardenne F‐51100 Reims France

**Keywords:** antibacterial, Fmoc‐FF, molecular dynamics, reactive oxygen species, siderophores

## Abstract

Injectable hydrogels are promising candidates as local drug delivery platforms for the treatment of infected wounds. Self‐assembled small peptide hydrogels are of interest due to their high biocompatibility, degradability, and ease of synthesis. This study describes the formation of an injectable hydrogel based on the self‐assembly of Fmoc‐FFpY (Fmoc: fluorenylmethoxycarbonyl, F: phenylalanine, pY: tyrosine phosphate) triggered by electrostatic interactions in the presence of Fe^3+^ ions. Stabilized by H bonding and π–π stacking, the hydrogels exhibit high mechanical stiffness with a *G*′ (storage modulus) of ≈8000 Pa and a self‐recovery up to *G*′ ≈100 Pa. Peptide self‐assembly yields β‐sheets twisted into fibrillar helices of 12 nm in diameter and pitch. Molecular dynamics simulations confirm 1) the aggregation of Fmoc‐FFpY in the presence of Fe^3+^ and the adopted secondary structure and show that 2) the aggregated Fmoc‐FFpY/Fe^3+^ disrupts the bacterial membrane of *Staphylococcus aureus* and *Pseudomonas aeruginosa*, favoring the passive entry of Fe^3+^ into the pathogen. In full agreement with the simulations, the hydrogels exhibit antibacterial activity against both bacteria, likely due to the increased Fe^3+^ entry into the cell, resulting in enhanced production of reactive oxygen species. This work paves the way for ferroptosis‐inducing treatment of bacterial infections using injectable ultrashort peptides.

## Graphical abstract

Triggered by electrostatic interactions and stabilized by H bonding and π–π stackings, Fmoc‐FFpY/Fe^3+^ self‐assembles into β‐sheets twisted into 12 nm diameter fibrillar helices. In full agreement with molecular dynamic simulations, the resulting injectable hydrogels have antibacterial activity by ferroptosis, enhanced production of reactive oxygen species due to the extensive cell entry of Fe^3+^ ions.

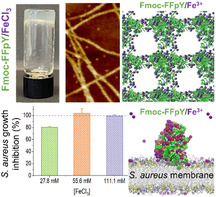

## 1 Introduction

Injectable hydrogels are gaining interest due to their mechanical similarity to natural tissues and high water content. This makes them promising candidates for biomedical applications such as minimally invasive surgeries for drug delivery or tissue engineering.^[^ ^1^ ^]^ Their interesting properties stem from their ability to be injected as a liquid and regain a gel‐like mechanical strength after in situ application. Low molar mass hydrogels especially those prepared using peptides show promising properties in the biomedical field, such as biocompatibility, biodegradability, and generally nonimmunogenicity.^[^ ^2^ ^]^ Their synthesis by solid‐phase peptide synthesis allows for precise sequence control, reproducibility, and high control over the dispersity. In addition, the process is already automated and therefore easily scalable. The peptide alphabet consists of 20 canonical amino acids with different functions and can be easily derivatized to improve the properties or add functionalities to the peptide backbone. Pioneering work has been done in the field of ultrashort hydrogelators (i.e., less than three amino acids). In particular, the groups of Gazit and Ulijn have demonstrated the ability of the Fmoc‐FF (Fmoc: fluorenylmethoxycarbonyl, F: phenylalanine) peptide to form amyloid fibrils^[^ ^3^ ^]^ and hydrogels, respectively.^[^ ^4^ ^]^ Due to solubility issues, peptide self‐assemblies are usually triggered by pH, temperature, and solvent change.^[^ ^5^ ^]^ The group of Bin Xu has introduced the concept of enzyme‐instructed self‐assembly (EISA) by using an enzyme to hydrolyze a water‐soluble peptide to trigger its self‐assembly.^[^ ^6^ ^]^ Inspired by this work, our group has developed an original Fmoc‐FFpY (pY: tyrosine phosphate) peptide to introduce the concept of localized EISA. Fmoc‐FFpY was enzymatically dephosphorylated using alkaline phosphatase (AP) to form the Fmoc‐FFY fibrillar hydrogel on a surface. AP, embedded in a polyelectrolyte multilayer, formed the “reaction motor” and a polyelectrolyte covalently modified by anchoring hydrogelator peptides, the top of the multilayer. The last layer acted as a nucleation site for the self‐assembly of the Fmoc‐FFY peptides, resulting in a nanofiber network.^[^ ^7^ ^]^ Fmoc‐FFpY can also form an injectable hydrogel when formulated with polycations,^[^ ^8^ ^]^ cationic polymer nanoparticles,^[^ ^9^ ^]^ or sodium ions, a monovalent cation.^[^ ^10^ ^]^ Several reports have investigated the self‐assembly of short peptides in the presence of salt^[^ ^11^ ^]^ attributing it to the salting‐out effect and charge screening.^[^ ^12^ ^]^

Peptide‐based hydrogels, which either inherently possess antimicrobial activity or contain antimicrobial agents, have shown promise in the treatment of localized infections with reduced systemic toxicity.^[^ ^13^ ^]^ Self‐assembled peptides, composed of arginine or lysine and hydrophobic amino acids, have been developed with inherent antibacterial activity due to the disruption of bacterial membranes.^[^ ^14^ ^]^ They are based on β‐hairpin,^[^ ^15^ ^]^ designed as cationic multidomain peptides (with a central amphiphilic part flanked by two positively charged ends),^[^ ^16^ ^]^ or as amphiphilic peptides, with one lysine or one arginine and a hydrophobic tail composed of several alanine.^[^ ^17^ ^]^ Ultrashort peptides FF (F: phenylalanine) formed nanotubes effective against bacteria, potentially by disrupting membranes.^[^ ^18^ ^]^ Following this work, Nap‐FFpY (Nap: naphthylacetic acid) was designed and able to cross the bacterial membrane to self‐assemble inside the bacteria, dephosphorylated by phosphatase, leading to bacterial death.^[^ ^19^ ^]^ We have shown that self‐assembled Fmoc‐FFY has a greater antibacterial activity than free Fmoc‐FFpY, suggesting a link between assembly and antibacterial efficacy.^[^ ^20^ ^]^ Self‐assembled ultrashort peptides can also deliver antibiotics^[^ ^21^ ^]^ or antibacterial silver nanoparticles^[^ ^22^ ^]^ to kill bacterial cells without causing significant systemic toxicity. Self‐assembled Ag^+^‐coordinated Fmoc‐amino acids have even been shown to have a superior bactericidal property than Ag^+^ ions in solution due to a sustained release of both ions and peptides.^[^ ^23^ ^]^

Ferroptosis is a newly discovered form of cell death characterized by high levels of iron accumulation and lipid peroxidation. It is an attractive tool for cancer treatment^[^ ^24^ ^]^ and is beginning to be considered to cure resistant bacterial infections.^[^ ^25^ ^]^ Fe^2+^‐loaded hyaluronic acid^[^ ^26^ ^]^ and alginate hydrogels^[^ ^27^ ^]^ have been shown to eliminate antibiotic‐resistant bacterial infections and accelerate wound healing in a murine model. The loading of Fe^3+^/ethylenediaminetetraacetic acid complexes into hyaluronic acid^[^ ^28^ ^]^ and alginate/hyaluronic acid^[^ ^29^ ^]^ hydrogels was undertaken to obtain bactericidal properties due to iron ions release. Fe^3+^ ions complexed with hinokitiol, a metal chelator derived from natural plants, have been shown to trigger the production of intracellular reactive oxygen species (ROS) resulting in bacterial cell death.^[^ ^30^ ^]^ This approach has been effective in treating wound infection in a murine model. Fe^3+^‐loaded lipid nanoparticles have shown similar properties.^[^ ^31^ ^]^ To the best of our knowledge, no antibacterial Fe^3+^‐coordinated ultrashort peptide‐based hydrogels have been reported in the literature.

**Figure 1:**
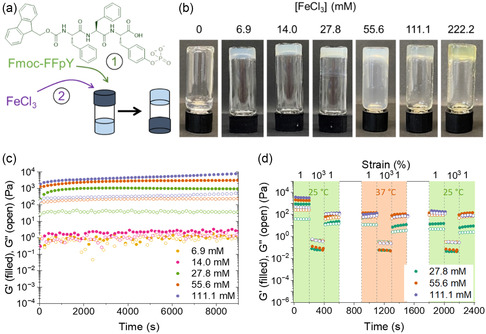
Macroscopic and viscoelastic properties of Fmoc‐FFpY/FeCl_3_ mixtures prepared at 5 mg mL^−1^ Fmoc‐FFpY and different salt concentrations. a) Schematic representation of Fmoc‐FFpY self‐assembly triggered by Fe^3+^ ions resulting in the formation of supramolecular hydrogels Fmoc‐FFpY/Fe^3+^. b) Inverted tube tests and c,d) rheological properties, storage modulus *G*′ (filled symbols), and loss modulus *G*″ (open symbols), as a function of time obtained in c) time sweep (0.01% strain, 1 Hz) at 25 °C and d) dynamic step strain amplitude test (1% or 1000% strain, 1 Hz) at 25 °C (green background) and 37 °C (orange background).

Following our previous physical chemistry study combining simulation and experiments on the self‐assembly of Fmoc‐FFpY in the presence of Na^+^, the present work aims to investigate experimentally and computationally the self‐assembly of Fmoc‐FFpY in the presence of the trivalent ion Fe^3+^ and to study the antibacterial activity of the resulting hydrogels (**Figure** **1**a). Hydrogels were formed thanks to electrostatic screening of the peptide and stabilized by the presence of H‐bonding and π–π stacking, shown experimentally and confirmed by molecular dynamic (MD) simulations. Fmoc‐FFpY/Fe^3+^ hydrogels exhibited high mechanical stiffness and injectable properties due to shear thinning behavior and good injectability. Peptide self‐assembly yielded β‐sheets twisted into fibrillar helices with J‐aggregates as revealed experimentally and computationally. Finally, the antibacterial activity of the hydrogels was evaluated both through MD simulations and in vitro against *Staphylococcus aureus* and *Pseudomonas aeruginosa*, gram‐positive and gram‐negative bacteria, respectively. Fmoc‐FFpY/Fe^3+^ hydrogels showed enhanced bactericidal properties due to the delivery of Fe^3+^ within the pathogen by the self‐assembled peptide, resulting in the lethal intracellular production of ROS. This combined experimental and computational study paves the way for ferroptosis‐inducing treatment of bacterial infections using injectable ultrashort peptides.

## 2 Results and Discussion

### 2.1 Formulation and Mechanical Properties of Fmoc‐FFpY/Fe^3+^ Self‐Assemblies

The inverted tube test was performed on Fmoc‐FFpY/FeCl_3_ mixtures after 24 h to assess the macroscopic formation of hydrogels. Stock solutions of Fmoc‐FFpY and FeCl_3_ in 0.1 M phosphate buffer (PB) at pH 7.4 were used to produce different mixtures with a final Fmoc‐FFpY concentration of 5 mg mL^−1^ (6.4 mM) and a final Fe^3+^ concentration varying from 0 to 222.2 mM. A viscous liquid is obtained at 0 mM FeCl_3_ and macroscopic hydrogels are formed for the other salt concentrations (Figure 1b). At 222.2 mM FeCl_3_, the hydrogel appeared yellow and was not investigated further. Fmoc‐FFpY/FeCl_3_ mixtures were characterized by oscillatory shear rheology to determine the storage modulus *G′* and the loss modulus *G*″. First, the evolution of the viscoelastic properties as a function of time after mixing was monitored using the small amplitude dynamic time sweep (Figure 1c). At 6.9 and 14.0 mM in FeCl_3_, Fmoc‐FFpY/FeCl_3_ mixtures have a liquid‐like behavior with *G*′ ≈ *G*″ around 1 Pa. At 27.8, 55.6, and 111.1 mM, the gelation occurs rapidly with *G*′ being several times higher than *G*″ and increasing slightly until reaching a plateau after 2000 s (33 min). Fmoc‐FFpY/Fe^3+^ hydrogels exhibit a high mechanical stiffness with a *G*′ ranging from 1000 to 8000 Pa for FeCl_3_ concentrations from 27.8 to 111.1 mM. For comparison, Fmoc‐FFpY/Na^+^ hydrogels have a *G*′ of 53 Pa at 500 mM NaCl.^[^ ^10^ ^]^ As previously reported for other peptides^[^ ^32^ ^]^ and Fmoc‐FFpY/Na^+^,^[^ ^10^ ^]^ increasing the salt concentration results in stiffer hydrogels. The viscoelastic properties of Fmoc‐FFpY/Fe^3+^ are similar to those of Fmoc‐FFpY/poly(allylamine hydrochloride) (PAH) hydrogels.^[^ ^8^ ^]^ All Fmoc‐FFpY/Fe^3+^ hydrogels are in the linear viscoelastic region below 1% strain with a gel‐to‐sol transition with increasing strain, resulting in a liquid solution at 1000% strain (Figure S1, Supporting Information). To evaluate the injectability of the hydrogels, strain sweep tests were performed at 25 and 37 °C on Fmoc‐FFpY/Fe^3+^ hydrogels prepared at 27.8, 55.6, and 111.1 mM FeCl_3_. Dynamic step strain amplitude tests were conducted by varying the strains between 1% and 1000% for short times to simulate the deformation caused by injection through a syringe (Figure 1d). At a strain of 1000%, the hydrogels exhibit liquid‐like behavior (*G*″ > *G*′) and regain gel‐like behavior with *G*′ greater than *G*″ when the strain was reduced to 1%. After the first deformation run, the stiffness of the hydrogels decreases. As a result, *G*′ ranges from 50 to 100 Pa for 27.8–111.1 mM FeCl_3_, maintaining a gel‐like behavior even after multiple deformations. The test was also performed at 37 °C and showed no temperature effect on the mechanical properties of the gels. In summary, Fmoc‐FFpY/Fe^3+^ hydrogels recovered gel‐like properties after high‐strain deformations resulting in viscoelastic properties close to those of the extracellular matrix^[^ ^33^ ^]^ making them suitable candidates as injectable hydrogels.

### 2.2 Morphological Characterizations of Fmoc‐FFpY/Fe^3+^ Self‐Assemblies

**Figure 2:**
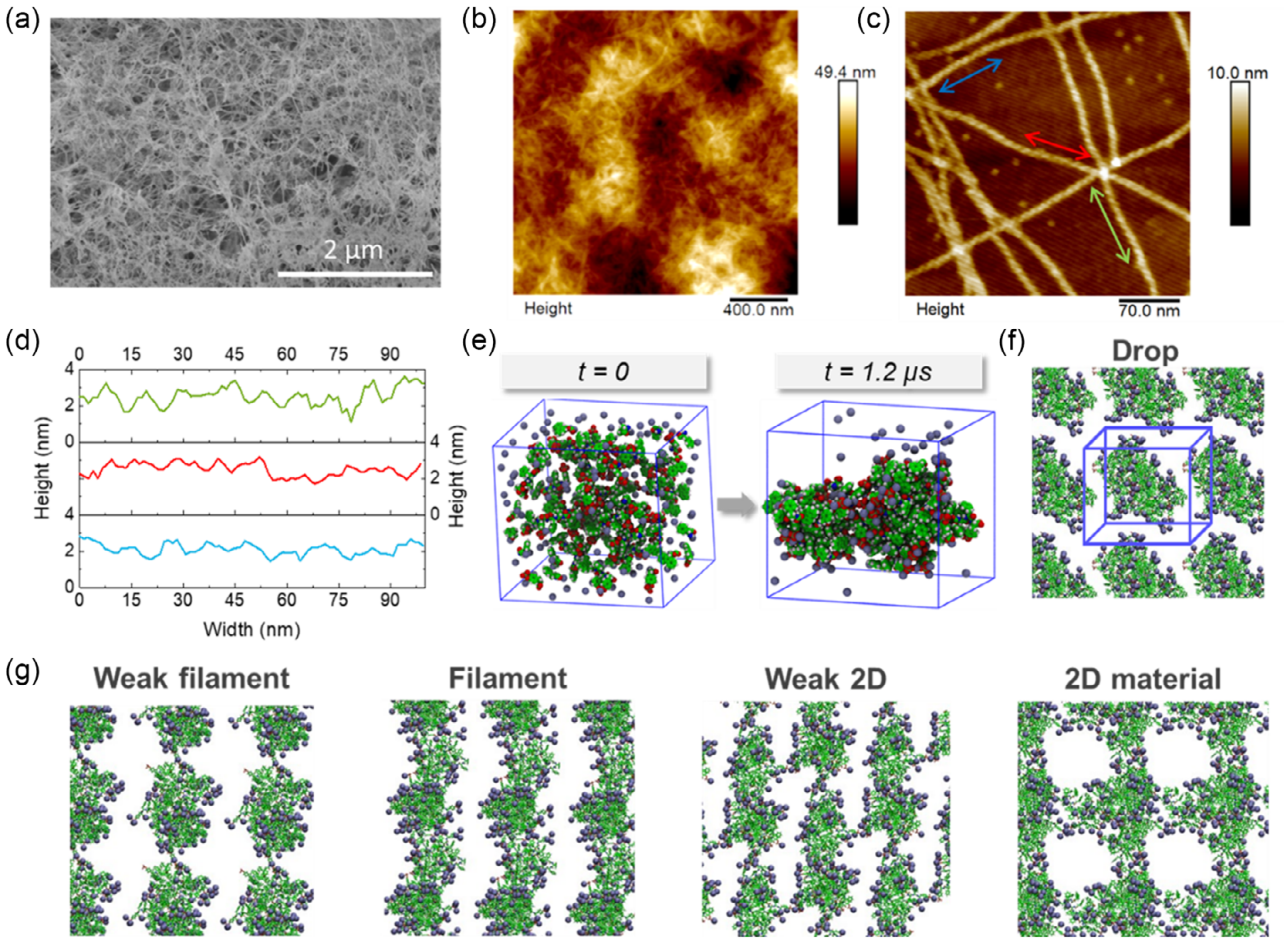
Morphology of Fmoc‐FFpY/Fe^3+^ self‐assembly: experimental and computational results. a) Cryo‐SEM image obtained at 27.8 mM FeCl_3_, typical AFM images in dry state in b) tapping mode at 14.0 mM, and c) peak force tapping mode at 27.8 mM FeCl_3_ after a tenfold dilution of the sample with three isolated single fibers marked by colored double arrows, and d) their respective longitudinal section showing a regular pattern. e) MD simulations with 40 Fmoc‐FFpY units (carbon, oxygen, and hydrogen are displayed with green, red, and white colors, respectively) randomly arranged in the presence of 120 FeCl_3_ (iron ions = blue spheres) at *t* = 0 and after 1.2 μs dynamics, and f) the peptide units assembled into drop form replicated in cubic unit. H_2_O molecules are hidden for better clarity. g) Illustrations of the various supramolecular organizations obtained in the simulation. Only the cations (Fe^3+^) close to 4 Å of peptides are displayed with ice‐blue van der Waals (vdW) spheres.

The morphology of Fmoc‐FFpY/Fe^3+^ self‐assemblies was characterized experimentally and computationally. Cryo‐scanning electron microscopy (cryo‐SEM) and atomic force microscopy (AFM) images reveal a dense network of fibers for samples prepared at 27.8 mM FeCl_3_ (**Figure** **2**a and Figure S2, Supporting Information) and 14 mM FeCl_3_ (Figure 2b), respectively.

The hydrogels at higher FeCl_3_ concentrations were too dense to be characterized. AFM analysis was performed on tenfold diluted samples deposited on a silicon wafer to image isolated fibrils of the Fmoc‐FFpY/Fe^3+^ mixture prepared at 27.8 mM FeCl_3_ (Figure 2c). Fibrils with a width estimated to 12 ± 2 nm, a height of 3.5 ± 0.5 nm, and a length of several micrometers are observed. The adsorption of the peptide on the silicon wafer likely induces a deformation of the fiber diameter. Similar fibril sizes were observed for Fmoc‐FFpY/P self‐assemblies and smaller ones for Fmoc‐FFpY/Na^+^.^[^ ^8^ ^]^ They have a helical structure with a periodicity of 12 ± 2 nm evaluated on the longitudinal section of isolated fibrils by measuring the mean value of the peak‐to‐peak distance (ten measurements) (Figure 2d). Fmoc‐FFF self‐assembly presents a helical structure with a periodicity of 17.4 ± 0.1 nm, which is very similar to our result.^[^ ^34^ ^]^ The Fmoc‐FFpY/Na^+^ has been reported to form bundles of fibers, i.e., large ribbons with a periodicity of 100 nm.^[^ ^35^ ^]^ The Fmoc‐FFpY/Fe^3+^ self‐assembly was further investigated by computational studies to obtain information at the atomic level. MD simulations were performed for 1.2 μs with 40 Fmoc‐FFpY peptides randomly placed in a confined space, at least 20 Å from each other to avoid any potential association due to proximity, in the presence of 120 FeCl_3_ and repeated 4 times to ensure the reproducibility of the results (Figure 2e). MD simulations show that the Fmoc‐FFpY peptide tends to self‐assemble. The calculation of non‐native contacts provided valuable information about the quick aggregation of Fmoc‐FFpY peptides, which typically occurs during the first 300 ns of simulation (Figure S3, Supporting Information). Figure 2f,g illustrates the different supramolecular organizations obtained for the system by replicating the confined space in the three spatial directions (Figure S4, Supporting Information). The statistical trend was extracted from 20 simulations (Table S1, Supporting Information). A low proportion of drops was found (10%), which can be attributed to peptide aggregates. Structuration in filaments (20% of weak filaments and 25% of filaments) and 2D materials (10% of weak 2D and 35% of 2D) were observed corresponding to the formation of a network of fibers. These results highlight the structuration of the peptide self‐assembly comforting the experimental results. Close observation of the trajectories reveals the role of Fe^3+^ ions, which screened the negative charges of the peptides by electrostatic compensation allowing them to approach each other over long distances. Subsequently, Fe^3+^ ions form bridges between the peptide units allowing stacking (J‐aggregates, see paragraph below). The role of Fe^3+^ ions is therefore not only a spectator but a direct actor in the hydrogel formation.

### 2.3 Secondary Structure and J‐Aggregates of the Fmoc‐FFpY/Fe^3+^ Self‐Assemblies

**Figure 3:**
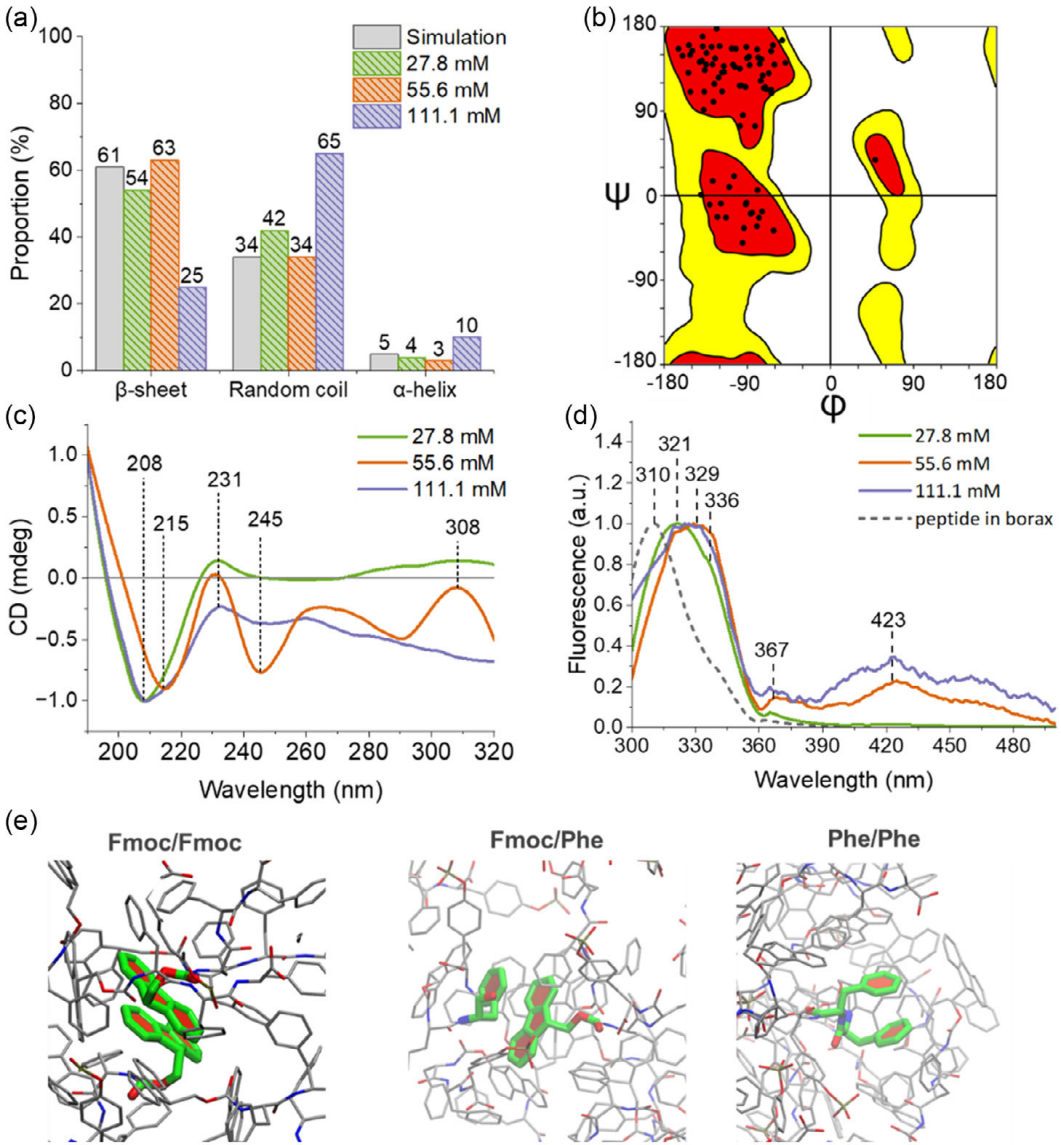
Secondary structure and J‐aggregates of Fmoc‐FFpY/Fe^3+^ self‐assembly. a) Amide I band contributions evaluated from ATR‐FTIR spectra and by MD simulation. b) Example of Ramachandran map obtained by MD simulation. c) CD spectra of the hydrated samples. d) Normalized fluorescence emission spectra (*λ*_EXC_ = 290 nm) of Fmoc‐FFpY in the presence of different FeCl_3_ concentrations and free peptide solubilized in borax (25 mM, pH 9.5). e) Examples of J‐aggregates recorded during MD simulations. From left to right, J‐aggregates between Fmoc/Fmoc, Fmoc/Phe, and Phe/Phe.

To gain more insight into the molecular interactions driving the peptide self‐assembly, the Fmoc‐FFpY/Fe^3+^ mixtures were characterized by spectroscopic methods. First, the secondary structure of the Fmoc‐FFpY/Fe^3+^ self‐assemblies was analyzed in the dry state by attenuated total reflection‐ Fourier‐transform infrared (ATR‐FTIR) and in the hydrated state by circular dichroism (CD). Figure S5 (Supporting Information) shows the region of the amide I band of the peptide (1600–1700 cm^−1^) of Fmoc‐FFpY/Fe^3+^ hydrogels prepared at 27.8, 55.6, and 111.1 mM FeCl_3_ with the peak at 1690 cm^−1^ attributed to the carbamate.^[^ ^36^ ^]^ The amide I band was decomposed to identify the contributions of the secondary structures adopted by the peptides.^[^ ^36^, ^37^ ^]^ **Figure** **3**a and Table S2 (Supporting Information) show the contribution of each peak area to the amide I band.

At 27.8 and 55.6 mM FeCl_3_, Fmoc‐FFpY/Fe^3+^ hydrogels contain similar secondary structure contributions: 53%–54% β‐sheets, 42%–44% random coils, and 3%–4% α‐helices. In contrast, at 111.1 mM FeCl_3_, the hydrogels have 65% random coils, 25% β‐sheets, and 10% α‐helices. A higher concentration of iron ions induces a less structured peptide self‐assembly with a small increase in the α‐helix contribution. Ramachandran plots were used to decipher the secondary structures of Fmoc‐FFpY supramolecular organizations obtained from MD trajectories. Figure 3b shows the Ramachandran map with β‐sheet (61.2 ± 1.7%), random coil (34.0 ± 1.6%), and α‐helix (4.9 ± 0.5%) structures was observed within the assemblies (Table S3, Supporting Information). In the MD simulation, the peptide‐to‐iron ratio was set to 0.3, close to the 27.8 mM FeCl_3_ with a molar ratio of 0.23 used in the experiments. The values of the proportions of secondary structures were reported in Figure 3a, showing that the MD simulations closely reproduce the experimental observations. This strongly validates the calculations and allows us to explore the different structural elements at the atomic scale with confidence. CD spectroscopy was used to analyze the structural arrangement of the hydrated samples prepared at 27.8, 55.6, and 111.1 mM FeCl_3_ (Figure 3c and Figure S6, Supporting Information). Fmoc‐FFpY/Fe^3+^ hydrogels show a decrease in signal from a positive value at 200 nm, suggesting the presence of a positive band below 190 nm, and a negative peak at 208 nm for 27.8 and 111.1 mM and at 215 nm for 55.6 mM FeCl_3_. These signals are attributed to the presence of β‐sheets,^[^ ^38^ ^]^ in agreement with the results obtained by ATR‐FTIR and MD simulations. Thus, Fmoc‐FFpY/Fe^3+^ self‐organizes into twisted β‐sheet structures that form single helical fibers as observed by AFM (Figure 2c). Previous research work suggested that Fmoc‐FF peptides can be arranged in antiparallel β‐sheets to maximize Fmoc overlap and that twisted β‐sheets do not alter the CD pattern of the peptide.^[^ ^39^ ^]^ In comparison, Fmoc‐FFpY/Na^+^ self‐assemblies have a helical secondary structure resulting in the helical twisting of the fiber bundle.^[^ ^10^ ^]^ On the CD spectra of the hydrogel prepared at 27.8 mM, the maxima at 231 and 308 nm are attributed to the stacking of the aromatic units of Fmoc‐FFpY and the offset face‐to‐face stacking of the fluorenyl absorption, respectively.^[^ ^8^ ^]^

Fluorescence emission spectrum analysis was conducted to investigate the π–π stacking between the fluorenyl chromophores. Figure 3d displays the fluorescence spectra of Fmoc‐FFpY/Fe^3+^ hydrogels compared to a non‐self‐assembled Fmoc‐FFpY solution, prepared in borax buffer pH 9.5. Nonstacked fluorenyl moieties have a maximum fluorescence emission at 310 nm. In the presence of iron salt, a redshift of the fluorescence emission is observed up to 321, 333, and 329 nm at 27.8, 55.6, and 111.1 mM FeCl_3_, respectively. A shoulder peak at 336 nm is observed indicating the presence of antiparallel Fmoc overlaps, with a Stokes shift of 2392 cm^−1^. For 55.6 and 111.1 mM FeCl_3_, corresponding to Fe^3+^:peptide molar ratio of 8.7:1 and 17.4:1, Fmoc‐FFpY/FeCl_3_ mixtures have another peak of fluorescence at 423 nm, which is a characteristic signal of J‐aggregates,^[^ ^40^ ^]^ with a Stokes shift of 8624 cm^−1^. This indicates the presence of densely packed antiparallel and parallel Fmoc units, allowing electrons to move and resulting in the appearance of this band.^[^ ^41^ ^]^ The formation of J‐aggregates was then investigated by MD simulations (Figure 3e and Table S4, Supporting Information). To the best of our knowledge, this is the first time that J‐aggregates have been studied from MD simulations for gelators, leading to the development of open‐source software for this task.^[^ ^42^ ^]^ 36% of the Fmoc‐FFpY peptides (15 over 40) are involved in various J‐aggregates (Fmoc/Fmoc, Fmoc/Phe, and Phe/Phe) (Figure 3e), in agreement with the experimental results. Interestingly, Fmoc‐FFpY/Fe^3+^ hydrogels with J‐aggregates have a *G*′ greater than 1000 Pa. J‐aggregates have been also observed in Fmoc‐FFpY/PAH hydrogels^[^ ^8^ ^]^ and other Fmoc‐based dipeptide^[^ ^40^ ^]^ which have similar viscoelastic properties.

### 2.4 Fmoc‐FFpY and iron ions Release from Fmoc‐FFpY/Fe^3+^ Hydrogels

**Figure 4:**
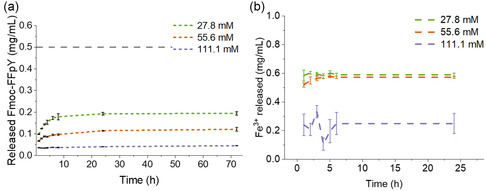
Release of Fmoc‐FFpY and Fe^3+^ from hydrogels in physiological medium. Cumulative amounts of a) Fmoc‐FFpY released in Mueller Hinton broth b) of Fe^3+^ ions released in physiological medium (Roswell Park Memorial Institute medium, RPMI) versus time. The grey dashed line represents the maximum Fmoc‐FFpY released assuming a complete dissolution of the hydrogel.

Formulated in 100 mM PB, the stability of the Fmoc‐FFpY/Fe^3+^ hydrogels in contact with a physiological medium (PB saline, PBS) was evaluated for 21 days at 37 °C by visual assessment (Figure S7, Supporting Information). The three formulated Fmoc‐FFpY/Fe^3+^ hydrogels were stable. The amounts of Fmoc‐FFpY and Fe^3+^ released in the physiological medium were measured by fluorescence, using the Fmoc emission of the isolated peptide at 310 nm and the quenching of calcein blue in the presence of Fe^3+^ at 431 nm, respectively (**Figure** **4**a,b). For the three hydrogels tested, a rapid release of Fmoc‐FFpY is observed up to 5 h followed by a slow release to a plateau reaching 0.195 ± 0.007, 0.121 ± 0.009, and 0.045 ± 0.001 mg mL^−1^ after 10 h for hydrogels prepared at 27.8 mM, 55.6 mM, and 111.1 mM FeCl_3_, respectively.

The mechanical properties of the hydrogels are consistent with these results since the softest hydrogel (*G*′ ≈ 1 kPa) prepared at 27.8 mM FeCl_3_ has a less dense network and tends to dissolve faster than the stiffest hydrogel (*G*′ ≈ 8 kPa) prepared at 111.1 mM FeCl_3_. Knowing that complete dissolution of the gels would lead to 5 mg mL^−1^ Fmoc‐FFpY (gray dashed line in Figure 4c), the peptide is not completely released even after 72 h. The release of Fe^3+^ ions is faster compared to the peptide reaching a plateau after 1 h at 0.58 ± 0.04, 0.52 ± 0.02, and 0.24 ± 0.08 mg mL^−1^ (2.16 ± 0.14, 1.92 ± 0.06, and 0.89 ± 0.28 mM) for hydrogels prepared at 27.8, 55.6, and 111.1 mM FeCl_3_, respectively (Figure 4d). In agreement with the network density, and similar to the peptide release, the hydrogels prepared with the lowest concentration in FeCl_3_ release the highest amount of iron ions and show the weakest stability.

### 2.5 Prediction of the Antibacterial Activity of Fmoc‐FFpY/Fe^3+^ Self‐Assembly by MD Simulations and in vitro Biological Assays

**Figure 5:**
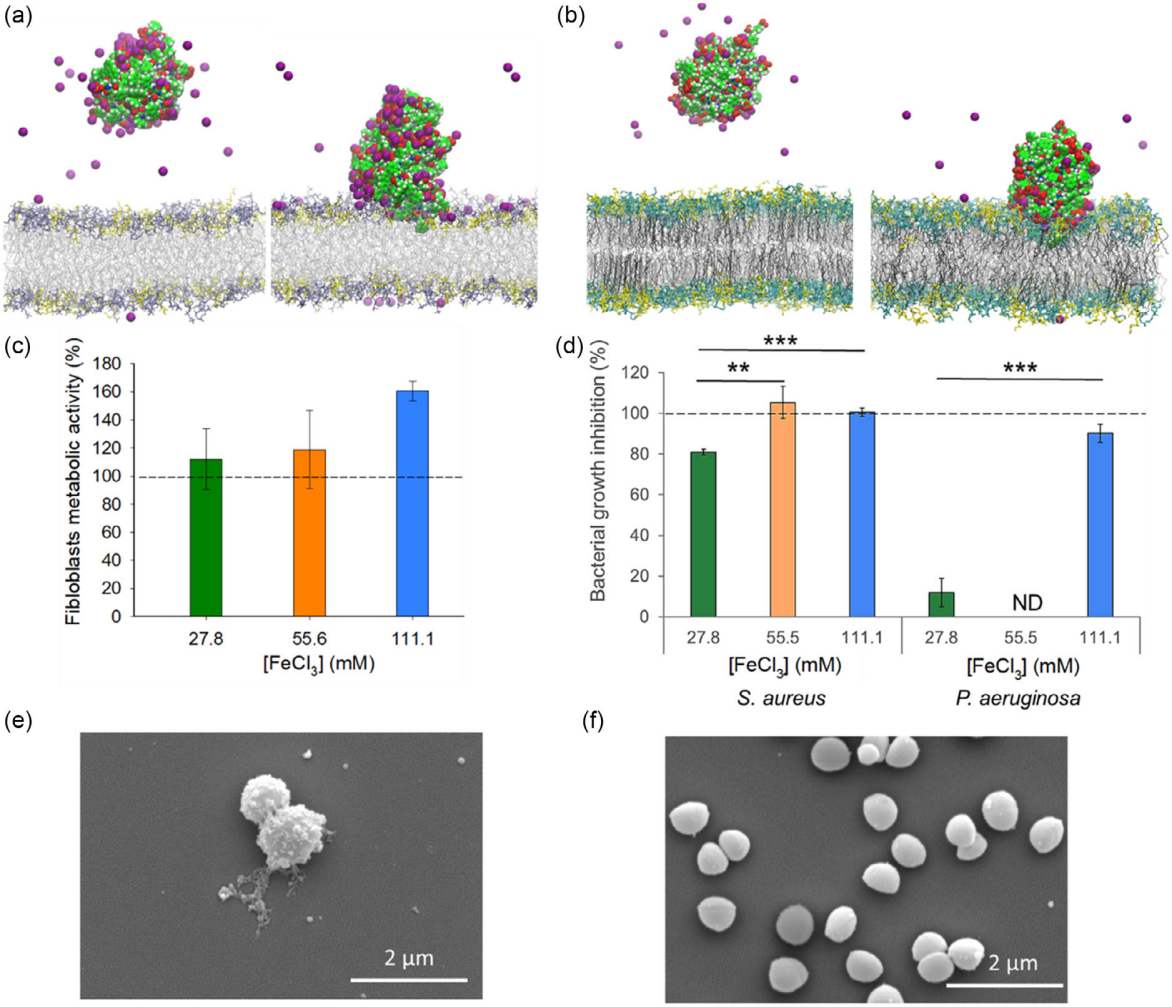
Prediction of the antibacterial activity of Fmoc‐FFpY/Fe^3+^ hydrogels by MD simulation and biological assays. a,b) Example of starting system before and after MD simulation of hydrogels in the presence of bacteria membranes: the lipidic compositions mimic the (a) *S. aureus* and (b) *P. aeruginosa* membranes with the lipidic parts (oleic and palmitic in grey and black lines, respectively) and the heads (phosphocholine, phospho‐*rac*‐(1‐glycerol) and phosphoethanolamine in blue, yellow and cyan colors, respectively). Fe^3+^ and the hydrogel are displayed with vdW spheres in purple and green carbon atoms, respectively. For the sake of clarity, water is removed from the representations. c) Metabolic activity of dermal human fibroblasts in the presence for 24 h of activated media, previously in contact with Fmoc‐FFpY/Fe^3+^ hydrogels (*n* = 6 no significant difference). d) Growth inhibition of *S. aureus* and *P. aeruginosa* in the presence of Fmoc‐FFpY/Fe^3+^ hydrogels (*n* = 18). The dashed lines correspond to the positive control, i.e., agar gel and antibiotic mixture (tetracycline and cefotaxime), and ND means not determined. ** and *** symbols are for *p*‐values of 0.01 and 0.001, respectively. e,f) SEM images of *S. aureus* after 24 h contact with e) the hydrogel formulated at 111.1 mM FeCl_3_ and f) 5 w/v% agar hydrogel.

Knowing that Fmoc‐FFpY and Fe^3+^ are reported to be antibacterial, a series of MD simulations were performed to predict the antibacterial activity of Fmoc‐FFpY/Fe^3+^ hydrogels by placing the hydrogel over a cytoplasmic membrane mimicking that of *S. aureus* and *P. aeruginosa*, gram‐positive and gram‐negative bacteria, respectively (**Figure** **5**a,b). The outer peptidoglycan layer surrounding the cytoplasmic membrane of gram‐positive bacteria, such as *S. aureus*, is a mesh permeable to drugs, allowing them to diffuse through and reach the membrane.^[^ ^43^ ^]^ These membrane models were recently validated through experimental and theoretical studies of antibacterial peptides targeting *S. aureus* and *P. aeruginosa*.^[^ ^44^ ^]^ Despite the overall anionic electrostatic potential of the membrane, the hydrogel structure is structurally stable and inserts itself into the membrane, as illustrated in Figure 5a,b. In particular, the hydrophobic groups of Fmoc‐FFpY are found to be deeply inserted into the lipidic part of the membrane bilayer, resulting in strong anchoring. The contact between the membrane and the hydrogel, both negatively charged, is physically possible if electrostatic compensation occurs. This role is played by Fe^3+^ ions, which screen the hydrogel charges and thus promote the association. Fe^3+^ ions maintain the structural integrity of the hydrogel and also facilitate its anchoring to the *S. aureus* and *P. aeruginosa* membrane.

To quantify the interaction of the hydrogel with *S. aureus* and *P. aeruginosa* membranes, several metrics were calculated over the last 200 ns of the four MD simulation replicas for both membrane systems. First, the contact surface area with each membrane was determined. A contact area of 1732 ± 244 Å^2^ was observed for *S. aureus*, while a value of 1878 ± 324 Å^2^ was measured for *P. aeruginosa*. These very similar values can be compared to those previously reported for a peptide with strong antimicrobial activity, which showed a contact area of 2223 ± 183 Å^2^ for *S. aureus* and 2166 ± 201 Å^2^ for *P. aeruginosa*.^[^ ^44^ ^]^ The similarity in the magnitude of these values suggests a potential antibiotic activity of Fmoc‐FFpY/Fe^3+^ against these bacteria.

Furthermore, variations in species densities were also determined and compared with control systems containing the same number of Fe^3+^ ions above both membrane systems, but without the hydrogel. Figure S8a and S9a (lines in black, Supporting Information) show that the membrane remains symmetrical in the sole presence of Fe^3+^, as expected. The Fmoc‐FFpY/Fe^3+^ hydrogel inserts up to 7 Å into the *S. aureus* membrane and up to 10 Å into the *P. aeruginosa* membrane, which is remarkable. It reaches the lipid region resulting in 1) a reduction in the density of the lipid layer and 2) an expansion of the lipid layer thickness, indicating significant membrane swelling (Figure S8b and S9b, Supporting Information) with a greater impact for *S. aureus* than for *P. aeruginosa*. The anisotropic distribution of Fe^3+^ ions, due to the presence of the hydrogel, distorts the polar headgroup distribution within the membrane as visualized by the phosphorus densities (Figure S8c,d and S9c,d, Supporting Information). The local membrane curvature was determined, with a value of 56.8 ± 20.4 degrees for *S. aureus* and 48.6 ± 11.6 degrees for *P. aeruginosa*, indicating a strong bacterial membrane deformation that induces stress, linked to the antibacterial activity. Another measure of membrane disruption consists of calculating the area per lipid, averaged over the trajectories. For the *P. aeruginosa* membrane, an area per lipid of 12.08 ± 0.09 Å^2^ was observed in the presence of Fe^3^^+^ without the hydrogel, increasing to 14.25 ± 0.01 Å^2^ upon hydrogel insertion. This variation, indicative of membrane swelling and thus disruption, was even more pronounced for *S. aureus*, with the area per lipid increasing from 12.27 ± 0.11 Å^2^ without the hydrogel (under the same iron concentration) to 18.36 ± 0.02 Å^2^ following hydrogel insertion. The deuterium order parameter (SCD) was utilized to assess the impact of hydrogel‐induced disruption within the lipid region (Figure S10, Supporting Information). For both bacterial membrane models, the hydrogel caused a decrease in order, indicating an increase in disorder associated with the disruption, which propagated deeply into the central region of the membrane, extending up to the 16th carbon of the lipid chains. All these results show the membrane disruption induced by aggregated Fmoc‐FFpY/Fe^3+^, which could be related to its antibacterial activity and the effect is more pronounced for *S. aureus* than *P. aeruginosa*. Another important observation is that the hydrogel acts as a reservoir of Fe^3+^ cations, demonstrating the role of the Fmoc‐FFpY hydrogel in ion vectorization.

To ensure that Fmoc‐FFpY/Fe^3+^ hydrogels are not cytotoxic, the metabolic activity of human dermal fibroblasts in contact with extracts of the hydrogels was assessed following the ISO 10993‐5 standard. The metabolic activity of human dermal fibroblasts was evaluated after 24 h of culture in the presence of activated media compared to the basal cell culture medium. The activated media were obtained by exposing basal cell culture media to the hydrogels for 24 h. No impairment of the cell metabolic activity was observed for the three hydrogels, with no statistical difference (Figure 5c). Finally, the antibacterial activity of Fmoc‐FFpY/Fe^3+^ hydrogels was tested in vitro against *S. aureus* and *P. aeruginosa* in a Mueller–Hinton (MH) medium (Figure 5d). At 55.6 and 111.1 mM FeCl_3_, Fmoc‐FFpY/Fe^3+^ hydrogels reduce *S. aureus* growth by 104% and 100%, respectively, compared to the control (i.e., 0.5 wt% agar gel), which is significantly higher compared to 27.8 mM FeCl_3_ (81%). In the case of *P. aeruginosa*, the bacteria growth is also more inhibited with Fmoc‐FFpY/Fe^3+^ 111.1 mM (90%) than with Fmoc‐FFpY/Fe^3+^ 27.8 mM (12%). A reduced inhibitory effect for *P. aeruginosa* was observed compared to *S. aureus*, in agreement with MD simulations. This result can be due to the difference in cell wall composition between gram‐negative and gram‐positive bacteria, affecting bacterial sensitivity to antibiotics, antimicrobial peptides, and other antimicrobials.^[^ ^45^ ^]^ SEM analysis allowed the assessment of the membrane integrity of *S. aureus* after 24 h contact with Fmoc‐FFpY/Fe^3+^ hydrogels, prepared at 111 mM FeCl_3_ compared to the control, i.e., agar hydrogel (Figure 5e,f and Figure S13, Supporting Information). Rare bacteria were observed with a rough surface covered by a substance, probably self‐assembled peptides. This is likely to have caused damage to the cell wall, which was not visible likely due to the embedding of bacteria in the substance. In comparison, numerous bacteria with smooth and intact cell walls were observed after contact with the agar gel.

### 2.6 Mechanism of the Antibacterial Property of Fmoc‐FFpY/Fe^3+^ Hydrogels

**Figure 6:**
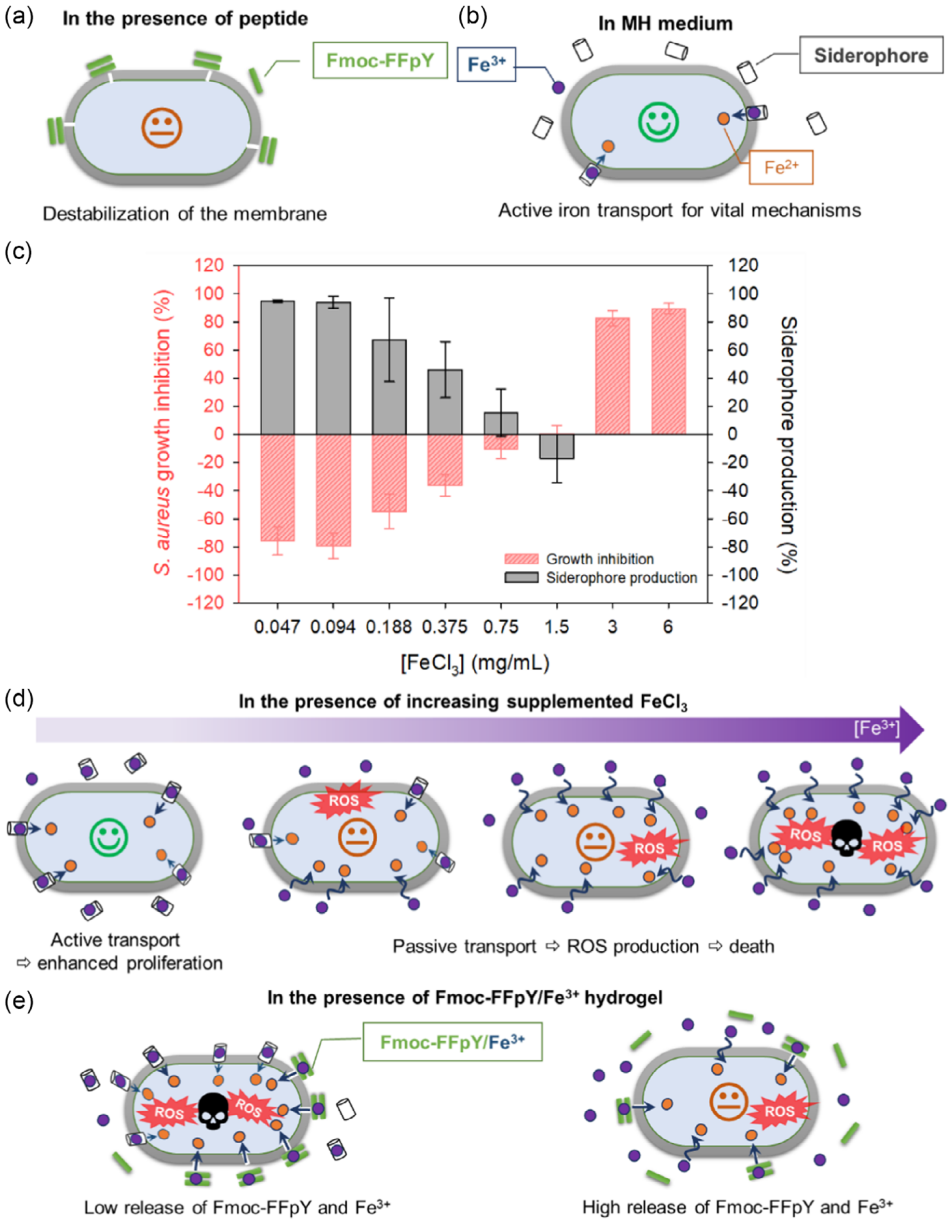
Antibacterial mechanism of Fmoc‐FFpY/Fe^3+^ hydrogels. Schematic representation of a) the antibacterial property of Fmoc‐FFpY in MH medium and b) siderophore active iron transport in bacteria cell to ensure vital mechanisms in MH medium. c) Production of siderophore (%) (*n* = 6) referred to without FeCl_3_ supplementation, and growth inhibition of *S. aureus* as a function of FeCl_3_ concentration (*n* = 18). Schematic representation of the effect on bacteria of d) supplemented Fe^3+^ ions: from better proliferation to death with increasing concentration and e) Fmoc‐FFpY/Fe^3+^ hydrogels (left) no or low peptide and iron ions release: combination of active (through siderophores) and passive (enhanced by self‐assembled Fmoc‐FFpY/Fe^3+^) iron entry in the cell leading to death and (right) at higher peptide and iron ions release: there is no production of siderophore and there is less self‐assembled Fmoc‐FFpY/Fe^3+^ leading to less entry of Fe^3+^.

Fmoc‐FFpY was reported to reduce *S. aureus* growth by 99% at 1 mg mL^−1^ in a cell culture medium (Roswell Park Memorial Institute [RPMI]).^[^ ^20^ ^]^ In MH medium, a slight antibacterial activity, i.e., 40% growth inhibition of *S. aureus*, was obtained at 1 mg mL^−1^ Fmoc‐FFpY (Figure S12a, Supporting Information). Self‐assembled in a physiological medium, as shown by the excimer formation (Figure S12b, Supporting Information), Fmoc‐FFpY peptides are likely prone to alter the cell wall integrity and further lead to bacterial death (**Figure** **6**a). Regarding Fe^3+^ ions, bacteria use them at low concentrations for vital mechanisms (DNA synthesis and repair, energy production, etc.). However, they become toxic at high concentrations.^[^ ^46^ ^]^ Siderophores, produced by bacteria, allow active uptake of Fe^3+^ ions at low concentrations (Figure 6b). They strongly bind Fe^3+^ ions and transport them to the cytoplasm, where they are reduced to Fe^2+^ ions in the presence of intracellular hydrogen peroxide (H_2_O_2_). At high concentrations, the toxicity of iron ions (transported passively) is attributed to the formation of ROS, through Fenton reactions catalyzed by Fe^2+^, which damage proteins and nucleic acids inside bacteria.^[^ ^47^ ^]^ In the presence of supplemented Fe^3+^ in the medium, the total amount of iron ions entering the cell could be the sum of those that have passed actively with siderophores and those that have passed passively.

The inhibitory effect of FeCl_3_‐supplemented medium on *S. aureus* growth was compared to that of pure MH medium (Figure 6c). Bacterial growth is inhibited by 90% at 3 mg mL^−1^, in contrast to concentrations ranging from 0.047 mg mL^−1^ to 0.750 mg mL^−1^, where the growth is favored (negative inhibition values). These results were compared to the siderophore production of *S. aureus* in the presence of FeCl_3_ (Figure 6c). Similar results have been reported in the literature for the same strain of *S. aureus*.^[^ ^48^ ^]^ At 0.05 mg mL^−1^, a high siderophore production accompanies a high bacterial proliferation. Above this value, an increase in FeCl_3_ concentration leads to a decrease in siderophore production by *S. aureus*, with a concomitant reduction of the bacterial proliferation toward the level in pure MH medium at 1.5 mg mL^−1^ FeCl_3_ (Figure 6c). At 3 and 6 mg mL^−1^ FeCl_3_, bacteria do not produce siderophores, but large amounts of Fe^3+^ are passively transported into the cell, leading to the lethal production of ROS^[^ ^47^ ^]^ (Figure 6d). *P. aeruginosa* bacteria also produce siderophores favoring the uptake of Fe^3+^ with an inverse dose–response relationship.^[^ ^49^ ^]^

The antibacterial activity of the Fmoc‐FFpY/Fe^3+^ hydrogels may be due to the simultaneous release of peptide and Fe^3+^ ions into the medium. However, the release of Fmoc‐FFpY is less than 0.2 mg mL^−1^ by the three hydrogels, which is insufficient to provide antibacterial activities above 80% (Figure 6a) and the release of Fe^3+^ is less than 0.4 mg mL^−1^, therefore likely to favor bacterial growth. Furthermore, the most bactericidal Fmoc‐FFpY/Fe^3+^ hydrogels release the lowest amount of both molecules, i.e., prepared at 55.6 and 111.1 mM FeCl_3_, which is the opposite of what would be expected (Figure 4c,d). Fmoc‐FFpY and Fe^3+^ ions must act synergistically within a certain concentration window. MD simulations predicted that self‐assembled Fmoc‐FFpY/Fe^3+^ would favor the passive entry of Fe^3+^ into the cell by altering the integrity of the bacterial cell wall (see Section 2.5). This passive entry will likely be added to the active entry by siderophores at a certain Fe^3+^ concentration window.

Prepared with 111.1 mM FeCl_3_, Fmoc‐FFpY/Fe^3+^ hydrogels, with 100% inhibition of *S. aureus* growth, release 1) 0.24 mg mL^−1^ Fe^3+^ maintaining the siderophore production, i.e., the active entry of Fe^3+^ into the cell, and 2) less than 1% of the Fmoc‐FFpY, resulting in efficient passive entry by the self‐assembled Fmoc‐FFpY/Fe^3+^ (Figure 6e, left). Thus, the bactericidal effect arises from the combined active and passive Fe^3+^ entry into bacteria, resulting in ROS formation, through Fenton reactions. Hydrogels prepared with 27.8 mM FeCl_3_ exhibit 81% inhibition of *S. aureus* growth, releasing 0.58 mg mL^−1^ Fe^3+^ and 45% of the Fmoc‐FFpY. Compared to hydrogels made with 111.1 mM FeCl_3_, bacteria do not produce siderophores, and there is a reduced amount of self‐assembled Fmoc‐FFpY/Fe^3+^ (Figure 6e, right). As a result, only a small fraction of Fe^3+^ can enter the cell through passive transport, leading to a reduced inhibitory effect on bacteria. In the case of hydrogels prepared with 55.6 mM FeCl_3_, we observed 104% inhibition of bacterial growth. This hydrogel releases a similar amount of Fe^3+^ as hydrogel prepared with 27.8 mM FeCl_3_. Thus, there is no siderophore production of *S. aureus*. Additionally, a smaller amount of Fmoc‐FFpY is released. As a result, a higher quantity of self‐assembled Fmoc‐FFpY/Fe^3+^ remains in contact with the bacteria, leading to increased passive iron entry and enhanced toxicity to the bacteria. This explains the counterintuitive observation that the most bactericidal Fmoc‐FFpY/Fe^3+^ hydrogels are those releasing the least peptide and iron ions. To further confirm the synergy between the peptide and iron ions, the antibacterial properties of Fmoc‐FFpY/Na^+^ hydrogels were evaluated, with the peptide acting as the sole antibacterial agent. Fmoc‐FFpY/Na^+^ self‐assembly was prepared with 250 mM NaCl, resulting in a hydrogel with a *G*′ (*G*″) of 20 Pa (9 Pa) (Figure S13a,b, Supporting Information). Releasing 0.459 ± 0.053 mg mL^−1^ Fmoc‐FFpY, the hydrogel demonstrated 72% inhibition of *S. aureus* growth (Figure S13c,d, Supporting Information). Higher bacterial growth inhibition was observed for the self‐assembled peptide compared to the same concentration in solution (Figure S12, Supporting Information), confirming our previous study.^[^ ^20^ ^]^ In comparison with the three Fmoc‐FFpY/Fe^3+^ hydrogels, the Fmoc‐FFpY/Na^+^ system releases between two‐ to tenfold the amount of Fmoc‐FFpY and is significantly less effective. The presence of Fe^3+^ plays a crucial role in enhancing the bactericidal properties of the self‐assembled Fmoc‐FFpY system.

## 3 Conclusion

We investigated experimentally and computationally the self‐assembly of Fmoc‐FFpY in the presence of a trivalent ion, Fe^3+^. Supramolecular hydrogels were formed with a helical fibrillar structure of 12 nm in diameter and pitch thanks to electrostatic compensation of the negative charges of the peptide by iron ions. β‐sheets and random structures were formed by the peptides as well as J‐aggregates. MD simulations confirmed the aggregation and the secondary structure of the peptides in the hydrogel. Fmoc‐FFpY/Fe^3+^ hydrogels exhibit high mechanical stiffness with a *G*′ (storage modulus) of ≈8000 Pa and self‐recovery properties up to *G*′ ≈ 100 Pa. MD simulations showed a peptide‐induced disruption of bacterial membranes and Fe^3+^ ions delivery of the self‐assembled peptide, which is likely related to its antibacterial activity. In full agreement with MD simulations, Fmoc‐FFpY/Fe^3+^ hydrogels exhibit antibacterial activity against *S. aureus* and *P. aeruginosa*, likely due to the enhanced intracellular production of ROS induced by the combined active and passive entry of Fe^3+^ ions. This experimental and computational study paves the way for ferroptosis‐inducing treatment of bacterial infections using injectable ultrashort peptides.

## 4 Experimental Section

4.1

4.1.1

##### Materials

Fmoc‐FFpY (<90%) was provided by Pepmic (Suzhou, China). Sodium phosphate monobasic monohydrate (>98%), sodium phosphate dibasic heptahydrate (ACS reagent, 98.0–102.0%), iron(III) chloride hexahydrate (≥99%), agar (98%), cefotaxime (≈95%), tetracycline (>95%), PBS, calcein blue (≤100%), and RPMI medium were purchased from Sigma–Aldrich. Sodium hydroxide (pellets, 99%) and hydrochloric acid (HCl) (37% p/p q. Soln.) were purchased from Thermo Fischer Scientific. MH broth medium was purchased from Medix. Sodium tetraborate anhydrous (98%) was purchased from Acros. All products were used as received.

##### Hydrogel Formation

Stock solutions of Fmoc‐FFpY (10 mg mL^−1^, 12.8 mM) and FeCl_3_ (13.8, 28, 55.6, 222.2, and 444.4 mM) were prepared in PB 0.1 M with the pH adjusted to 7.4. Different solutions were obtained by adding FeCl_3_ stock solution in Fmoc‐FFpY solution at a 1:1 volume ratio under stirring followed by centrifugation for 1 min at 9000 rpm to remove the trapped air bubbles. In each mixture, the final concentration of Fmoc‐FFpY was fixed at 5 mg mL^−1^ (6.4 mM) and the FeCl_3_ concentration varied between 0 and 222 mM. The final hydrogel volume was 200 μL for the inverted tube and was turned upside down after 24 h.

##### Fluorescence Spectroscopy

Fluorescence measurements were carried out using a plate reader (SAFAS Xenius XM). 100 μL of gel was placed in a 96‐well plate. The excitation wavelength was set to 290 nm and the emission spectrum was recorded between 300 and 600 nm. The photomultiplier tube voltage was adjusted for each sample and the emission intensity was normalized to compare the maximum emission wavelength of the varying gels.

##### Cryo‐SEM

A tiny piece of gel was quickly dipped into liquid ethane and then placed under a high vacuum (10^−6^ mbar) and low temperature (−150 °C) into the cryo‐preparation chamber (Quorum PT 3010) attached to the microscope. There, an adapted razor blade was used to fracture the frozen sample. To reveal the details of the morphology, it was slightly sublimated at −90 °C. Eventually, the sample was transferred to the Field Emission Gun‐cryo‐SEM (Hitachi SU8010) and observed at 1 kV at −150 °C.

##### AFM

AFM samples were prepared by putting a 30 μL drop of gel on a 1 × 1 cm^2^ silicon wafer that was previously cleaned with UV/ozone (UV/ozone ProCleaner BioForce Nanosciences) for 2.5 min. The hydrogel was spin‐coated using a Laurell WS‐650MZ‐23NPP/A3/AR3 spin coater (9000 rpm, 500 rpm acceleration). A drop of water was deposited over the dry gel and set for 30 s to remove the excess salt. The sample was again spin‐coated to remove the water and this process was repeated 3 times. Tapping mode AFM was performed on a Bruker AFM Multimode 8 (Bruker, USA) with a Nanoscope V controller and silicon cantilevers (resonance frequency 300 kHz, nominal spring constant of 42 N m^−1^, and 7 mm nominal tip radius). For the high‐resolution images, peak force tapping mode AFM was performed with another AFM probe (SCANASYST‐AIR‐HPI Bruker probe, nominal spring constant of 0.25 N m^−1^, and 2 nm nominal tip radius). The high‐resolution image showed visible stripes in the background due to electronic noise. We have deliberately presented the image with minimal processing (limited to planarity correction) to avoid data loss. The reported diameter corresponds to the width of the fiber calculated from a measurement on the AFM image, subtracted by 3 nm due to the roughness of the sample, which could potentially affect the accuracy of the tip.

##### FTIR Spectroscopy

200 μL of the gel were freeze‐dried overnight in a microvial of 1.5 mL and analyzed on an infrared spectrophotometer (Bruker FTIR Vertex 70) using ATR diamond. The absorbance spectrum was measured between 1500 and 1800 cm^−1^ with a 2 cm^−1^ step. The spectrum was an average of 288 scans. Deconvolution of the amide I band was performed using OPUS 7.5 software (Bruker Optik GmbH). First, the spectrum was cut between 1600 and 1700 cm^−1^. The baseline was then corrected and the curve was normalized using a “min–max” method. The frequencies of the peaks in the band were determined using the minimum positions of the second derivative. The number of smoothing points used to calculate the second derivative was adjusted between 9 and 25 depending on the noise of the curve. The spectrum was then fitted with Gaussian band profiles using 10 s Levenberg–Marquart's auto‐fit. The fitting quality was estimated by looking at the residual RMS error provided by the software. The relative contribution of each structure to the amide I band was calculated by the ratio of the area under each peak over the area of the total amide I band.

##### CD

After 24 h of gelation, 10 μL of gel was placed in a 0.01 mm path quartz cuvette. Spectra were recorded on a CD spectrophotometer (Jasco J1700) between 190 and 320 nm. The data pitch was 0.2 nm, CD scale 20 mdeg, D.I.T. 0.5 s, and bandwidth 10 nm. Five accumulations were performed for each spectrum. The spectrum was then smoothed with the Savitzky‐Golay 25 points method.

##### Rheological Characterization

Rheological properties were measured using a rheometer (Discovery HR20, TA Instruments) with a sand‐blasted plate/plate geometry of 20 mm in diameter and 0.5 mm gap. To determine the linear regime of the gel, an amplitude was performed for each gel by fixing the frequency at 0.1 Hz and varying the oscillation strain from 0.001% to 100%. To prepare the gel, and follow the gelation time, the mixing of the gel was performed directly on the rheometer. To do so, two drops of 42 μL Fmoc‐FFpY stock solution (10 mg mL^−1^) and two drops of 42 μL salt stock solution were placed on the plate and mixed at the beginning of the experiment by applying a stress growth of 10 s at 200 s^−1^, followed by 10 s at −200 s^−1^. The gelation was evaluated by time sweep over 2 h with a strain of 0.1% and a frequency of 1 Hz, at 25 °C. The injectability property of the gel was assessed by recording time sweeps at 1 Hz at a strain of 1% over 300 s followed by 1000% over 300 s, and finally 1% over 300 s again at 25 °C. The same steps were repeated at 37 °C, and again at 25 °C with 300 s between each temperature change to allow the gel to reach the right temperature.

##### Release of the Peptide

10 μL of 24 h old hydrogel was added to the well of a 96‐well plate. For each hydrogel, eight wells were filled to monitor the release kinetics. Each well corresponded to a specific time point. After 1 h, 190 μL of MH broth medium was added to each well, covered with an adhesive cover to prevent evaporation and incubated without stirring at 37 °C in an incubator. At various time intervals (1, 2, 3, 4, 6, 8, 24, and 72 h), 100 μL of the supernatant was withdrawn and placed in another 96‐well plate. To evaluate the amount of Fmoc‐FFpY released in the medium, the fluorescence emission intensity was recorded at 310 nm using a plate reader (SAFAS Xenius XM) with an excitation at 290 nm. The intensity was correlated with the quantity released using a calibration curve of Fmoc‐FFpY in MH.

##### Release of Fe^*3+*^

10 μL of 24‐hour‐old hydrogel was added in the well of a 96‐well plate. For each hydrogel, eight wells were filled to monitor the release kinetics. Each well corresponded to a specific time point. After 1 h, 190 μL of RPMI medium was added in each well, covered with an adhesive cover to prevent evaporation, and incubated without stirring at 37 °C in an incubator. At various time intervals (1, 2, 3, 4, 6, 24, and 27 h), 100 μL of the supernatant was withdrawn and placed in another 96‐well plate. 100 μL of a 1 mg mL^−1^ calcein blue solution was added. After stirring, the plate was allowed to stand at r.t. overnight. To evaluate the amount of Fe^3+^ released in the medium, the intensity of the fluorescence emission of calcein blue was recorded at 431 nm using a plate reader (SAFAS Xenius XM) with an excitation at 350 nm. The intensity was correlated with the quantity released using a calibration curve of various concentrations of Fe^3+^ with a 1 mg mL^−1^ concentration of calcein blue in RPMI.

##### Cytotoxicity Assays

Dermal human fibroblast harvesting was approved ethically and methodologically by our local Research Institution and was conducted with informed patients (written consent) following the usual ethical legal regulations (Article R 1243–57). All procedures were done following our authorization and registration number DC‐2014−2262 given by the National ‘‘Cellule de Bioéthique”. The freeze‐dried hydrogels (including a 5 wt% in PB agar hydrogel used as a control) were subjected to UV sterilization after which they were placed in the culture medium at 1 mg mL^−1^. The culture medium was incubated at 37 °C for 24 h, centrifuged to remove the hydrogel samples, and then placed in contact with the dermal human fibroblast cells for 24 h. A WST‐1 metabolic activity assay was then performed. For the WST‐1cell viability assay (Roche Diagnostics, Meylan, France), the absorbance was measured at 440 nm (A_440_) using a FLUOstar Omega microplate reader (BMG Labtech, Ortenberg, Germany) against a background control as blank. A wavelength of 750 nm was used as a correction. The metabolic activity was calculated using Equation (1). Each condition was performed in triplicate on three different hydrogels using the cells from two donors.

(1) $$\text{Metabolic activity}\left(\%\right)=100\times \frac{A_{S}}{A_{\text{control}}}$$

where $A_{\text{control}}$ is the A_440_ of the control and $A_{S}$ is the A_440_ of the solution prepared with Fmoc‐FFpY/Fe^3+^ hydrogel.

##### Preculture of Bacteria

Antibacterial assays were carried out on gram‐positive bacteria (*S. aureus*, ATCC 25923) and gram‐negative bacteria (*P. aeruginosa*, ATCC 27 853). Bacteria were precultured in aerobic conditions at 37 °C in MH broth medium at pH 7.4. A colony from a previously prepared MH/agar (BD BACTO) dish by spread plate method was transferred to 6 mL of MH medium and incubated overnight at 37 °C with stirring. A control was incubated in the same conditions without bacteria. The absorbance of the *S. aureus* or *P. aeruginosa* culture was measured at 600 nm (OD_600_) (Bio‐rad SmartSpec Plus UV/vis Scanning Photodiode Array Spectrophotometer) and adjusted to 0.001 by diluting in MH broth medium.

##### Antibacterial Assay of the Solutions

To evaluate the antibacterial activity of Fmoc‐FFpY in solution, the peptide was dissolved at 10 mg mL^−1^ in borax buffer, previously filtered over a sterile 0.22 μm mixed‐cellulose ester membrane, and lower concentrations were prepared by serial dilution in borax buffer. FeCl_3_ was dissolved at 120 mg mL^−1^ in PB, previously filtered over a sterile 0.22 μm mixed‐cellulose ester membrane, and lower concentrations were prepared by serial dilution in PB. 10 μL of each dilution was put in each well of a 96‐well plate and 90 μL of *S. aureus* culture (OD_600_ = 0.001) was added. 10 μL of MH and 90 μL of *S. aureus* culture were added to the control wells. Another control was prepared with 10 μL of antibiotics (0.1 μg mL^−1^ of tetracycline and 0.001 μg mL^−1^ of cefotaxime) and 90 μL of *S. aureus* culture. The plate was incubated for 24 h at 37 °C with stirring. The optical density at 600 nm (OD_600_) was then read using a multiplate reader (Accuris MR‐9600). The inhibition of *S. aureus* growth was calculated using Equation (2) and performed in sextuplet within each experiment and in triplicate for each tested species.

(2) $$\text{Growth inhibition }\left(\%\right)=100\times \frac{{\text{OD}}_{C=0}-{\text{OD}}_{C_{i}}}{{\text{OD}}_{C=0}}$$

where ${\text{OD}}_{C=0}$ is the OD_600_ of the control without peptide or antibiotics, and ${\text{OD}}_{C_{i}}$ is the OD_600_ of the solution with the concentration C_i_ of peptide or FeCl_3_.

##### Antibacterial Assays on Fmoc‐FFpY/Fe^*3+*^ *Hydrogels*

10 μL of 24 h old gel was placed in a well. As controls, 5 wt% agar hydrogels were prepared using a microwave in PB or an antibiotic mix (0.1 μg mL^−1^ of tetracycline and 0.001 μg mL^−1^ of cefotaxime). Gels were allowed to stand for 1 h after which 90 μL of *S. aureus* or *P. aeruginosa* culture (OD_600_ = 0.001) was added. It was incubated at 37 °C overnight without stirring. A control well was incubated in the same conditions without bacteria. The OD_600_ was then read using a multiplate reader (Accuris MR‐9600). The inhibition of bacterial growth was calculated with Equation (1) where ${\text{OD}}_{C=0}$ is the OD_600_ of the control with agar in PB, and ${\text{OD}}_{C_{i}}$ is the OD_600_ of the hydrogel prepared with a concentration $C_{i}$ of FeCl_3_, and was performed in sextuplet within each experiment and in triplicate for each tested species.

##### Chrome Azurol Assay

The pre‐chrome azurol assay (CAS) solution was prepared by adding a solution of 1.5 mL FeCl_3_ (1 mM) and 7.5 mL de CAS (2 mM) in HCl (10 mM) to 6 mL of hexadecyltrimethylammonium bromide (10 mM) under shaking. This solution was supplemented with 34.75 mL of anhydrous piperazine solution (4.307 g in distilled water) and its pH was adjusted to 5.6 with HCl 37%. The CAS solution was finally obtained by completing to 100 mL with distilled water. To evaluate the production of siderophores , *S. aureus* cultures were prepared with different concentrations of FeCl_3_ as described in the subsection Antibacterial Assay of the Solutions. Bacteria were removed from the 24 h culture by using sterile 0.22 μm mixed‐cellulose ester membranes, and Fe^3+^ was removed from the cultures by adding Cuprisorb (purchased by Seachem), a metal chelator, at 50 mg mL^−1^. After 24 h at 37 °C under stirring, Cuprisorb was removed by filtration with 0.22 μm mixed‐cellulose ester membranes. A 100 μL of the filtered solution was mixed with 100 μL of CAS solution, incubated for 3 h at 37 °C in the dark and the absorbance at 630 nm (A_630_) was read using a spectrophotometer (Varioskan LUX). The production of siderophores with Fe^3+^ supplementation was calculated with the following Equation (3) and normalized to without supplementation. The assay was performed in triplicate within each experiment and reproduced twice.

(3) $$\text{Siderophore production}\left(\%\right)=100\times \frac{A_{\text{CAS}}-A_{S}}{A_{\text{CAS}}}$$

where $A_{\text{CAS}}$ is the A_630_ of the CAS solution and $A_{S}$ is the A_630_ of the solution prepared from the bacterial culture supplemented with FeCl_3_.

##### SEM

SEM (FEI Quanta FEG250) was used to observe the morphology of *S. aureus* after 24 h proliferation assay in the presence of the hydrogels. After washing by centrifugation (1800 rpm) and resuspension in fresh PBS 3 times, 30 μL of bacterial suspension was deposited on a 12 mm diameter glass slide, incubated for 2 h at room temperature, and removed. 500 μL of glutaraldehyde 2.5% was deposited on the glass slide and removed after 15 min at room temperature to fix the bacterial cells, followed by washing 3 times with PBS, 3 times with water, and by consecutive 5 min immersion in ethanol‐in‐water baths (10, 25, 50, 70, 95, and 100% v/v 3 times). The samples were treated with hexamethyldisilazane (HMDS)/ethanol mixture (50:50 v/v) for 5 min followed by two consecutive incubations with the pure HMDS (5 min each). After removal of the HMDS solution, the samples were dried under a laminar flow fume hood and metalized with gold using a sputter coater before SEM observations.

##### MD—Creation and Parametrization of Molecules

All amino acids along with the phosphorylated residues were generated using the ff99SB force field^[^ ^50^ ^]^ of the Amber 22 software package.^[^ ^51^ ^]^ The Fmoc residue and florfenicol molecule were first built with the Maestro software^[^ ^52^ ^]^ and their partial atomic charges were determined through the semiempirical quantum mechanics computations AM1‐BCC.^[^ ^53^ ^]^ Phosphorylated tyrosine was further parametrized to become a C‐terminal residue by adding supplemental oxygen with an electron delocalization conducting to a carboxylate negative ion. Fmoc was considered as a residue for which connection rules were conducted with the LEap software^[^ ^51^ ^]^ leading to the Fmoc‐FFpY peptide assembly.

##### MD—Building Systems

The compaction of multiple units of Fmoc‐FFpY was the subject of our investigation by varying several initial conditions. In pursuit of this objective, five different systems were generated with the packmol software.^[^ ^54^ ^]^ 40 units of Fmoc‐FFpY were randomly distributed within a cubic space measuring 70 × 70 × 70 = 343 000 Å^3^. This method facilitated the generation of a diverse set of systems, each representing a unique configuration of the molecular units under study. Ions and water (TIP3P^[^ ^55^ ^]^) molecules were then added to all systems to account for periodic boundary conditions. Due to the limitations of the simulation box to accommodate varying concentrations, the molar ratio between Fmoc‐FFpY and FeCl_3_ was chosen as the primary parameter for comparing simulation results with experimental data. In the MD simulation, the peptide‐to‐iron ratio was set to 0.3, close to the 27.8 mM FeCl_3_ with a molar ratio of 0.23 used in the experiments.

##### MD—Simulations and Analyses Protocol

All simulations started with 400 000 steps of energy minimization with 100 000 steps of steepest descent followed by 300 000 of conjugated gradient algorithms.^[^ ^56^ ^]^ Systems were then heated to 300 K for 100 ps in the constant volume and temperature (NVT) ensemble and then switched to constant temperature and pressure (NTP) condition, through Berendsen weak coupling algorithm,^[^ ^57^ ^]^ for 500 ps to reach a stable temperature of 300 K using the Langevin thermostat with a collisional frequency of 1 ps^−1^.^[^ ^58^ ^]^ The nonbonded cutoff was set at10Å while the Particle Mesh Ewald method was used to treat long‐range electrostatic interactions.^[^ ^59^ ^]^ All these classical choices were made according to classical recommendations for obtaining trajectories at the microsecond scale.^[^ ^60^ ^]^ Hydrogen mass repartition^[^ ^61^ ^]^ was then used to employ a time‐step of 4 fs during the MD productions. MD trajectories, in the NTP ensemble, were engaged for 1.2 μs for all systems. For each system, four replicates were realized providing a statistical view of each considered system. This means that the system has five different starting conditions generated by packmol and for each four MD simulations were performed leading to a conformation time exploration of 1.2 × 20 = 24.0 μs. MD simulations involving interaction with a membrane were conducted using the Amber lipid21 force field.^[^ ^62^ ^]^ In this setup, the hydrogel structures were initially placed 25 Å above the membrane surface, ensuring no interaction occurred before the start of the simulation. For the *S. aureus* membrane, a peptide ratio of 70% 1,2‐dioleoyl‐*sn*‐glycero‐3‐phosphocholine and 30% 1,2‐dioleoyl‐*sn*‐glycero‐3‐phospho‐rac‐(1‐glycerol) was selected while, for *P. aeruginosa* membrane, a lipid ratio of 23% 1,2‐dipalmitoyl‐*sn*‐glycero‐3‐phosphoethanolamine, 46% of 1,2‐dioleoyl‐snglycero‐3‐phosphoethanolamine, and 31% of 1,2‐dipalmitoyl‐*sn*‐glycero‐3‐phosphoglycerol (DPPG) was generated according to previous studies.^[^ ^44^ ^]^ Structural analyses were obtained with the cpptraj module of AmberTools^[^ ^63^ ^]^ while VMD software^[^ ^64^ ^]^ was employed to visualize MD trajectories and make figures. Unless explicitly stated otherwise, the presented values are averaged across the simulation's replicas. An intermolecular contact is defined if a distance between heavy atoms is lower than 4 Å. Intermolecular contacts between Fmoc‐FFpY are qualified as weak if their number of contacts is between one to four and it is considered as classical if its number is up or equal to five. The radius of local curvature was computed and averaged along the trajectories with the memb‐curve software.^[^ ^42^ ^]^ A hydrogen bond is defined when the distance between the heavy atom's donor and acceptor of a hydrogen bond is lower than 3.4 Å and when the angle between donor, H, and acceptor atoms is lower than 60°.

##### Statistical Analysis

Outliers in OD_600_ raw data of the antibacterial assays were defined as more than 3 times the mean and were removed from the dataset (less than 1% of the values). The A_440_, the OD_600_, and CAS raw data were transformed to metabolic activity with Equation (1), inhibition of bacterial growth with Equation (2), and siderophore production with Equation (3), respectively, before calculation of the mean and standard error of mean of each experimental condition. The results of the cytotoxic, antibacterial, and CAS assays are presented as the mean standard error of the mean. Sample sizes (*n* = 6 for the cytotoxic assay, *n* = 18 for the antibacterial assays, *n* = 6 for the CAS assay) are specified in figure captions. The statistical analysis was performed using RStudio software (2024.12.0 build 467). The normality of the preprocessed data set was tested with a Shapiro–Wilk test followed by a Student (if normality) or Wilcoxon Mann–Whitney (if no normality) to assess the significant differences between the different conditions (two‐sided testing). The α‐level of significance was set at 0.05.

## Conflict of Interest

The authors declare no conflict of interest.

## Author Contributions

**Capucine Loth**: conceptualization (supporting); funding acquisition (equal); investigation (lead); methodology (lead); validation (equal); visualization (lead); writing—original draft (lead); writing—review editing (supporting). **Florent Barbault**: data curation (lead); investigation (equal); resources (supporting); software (lead); visualization (equal); writing—review editing (supporting). **Cécile Guégan**: investigation (supporting). **Flora Lemaire**: investigation (supporting). **Christophe Contal**: investigation (supporting). **Alain Carvalho**: investigation (supporting). **Sophie Hellé**: investigation (supporting). **Marie Champion**: investigation (supporting). **Halima Kerdjoudj**: supervision (supporting). **Delphine Chan‐Seng**: supervision (supporting); writing—review editing (supporting). **Lydie Ploux**: formal analysis (supporting); methodology (supporting); resources (supporting); supervision (supporting); validation (equal); visualization (supporting); writing—review editing (supporting). **Fouzia Boulmedais**: conceptualization (lead); funding acquisition (equal); project administration (equal); supervision (lead); validation (equal); visualization (supporting); writing—review editing (equal).

## Supporting information

Supplementary Material

## Data Availability

The data that support the findings of this study are available from the corresponding author upon reasonable request.
