# Supplementary material for: Experimental and Computational Study of Injectable Iron(III)/Ultrashort Peptide Hydrogels: A Candidate for Ferroptosis‐Induced Treatment of Bacterial Infections
Source: Small Sci. 2025 Apr 17;5(6):2400618. doi: 10.1002/smsc.202400618 (PMC12168595; doi:10.1002/smsc.202400618)
Supplement: Supplementary file 1 — Supplementary Material [file SMSC-5-2400618-s001.pdf]

Supporting Information

Experimental and Computational Study of Injectable Iron(III)/Ultrashort Peptide Hydrogels: A Candidate for Ferroptosis-Induced Treatment of Bacterial Infections

*Capucine Loth, Florent Barbault, Cécile Guégan, Flora Lemaire, Christophe Contal, Alain Carvalho, Sophie Hellé, Marie Champion, Halima Kerdjoudj, Delphine Chan-Seng, Lydie Ploux, and Fouzia Boulmedais**

1. Rheology 2
2. Cryo-SEM images of Fmoc-FFpY/Fe^{3+} hydrogels 2
3. Molecular dynamics (MD) simulations: aggregation and supramolecular organization 3
4. Infrared spectroscopy and MD simulation of the secondary structure 5
5. Circular dichroism 7
6. Molecular dynamics (MD) simulations: J-aggregates 8
7. Stability of the Fmoc-FFpY/Fe^{3+} hydrogels in contact with PBS 9
8. MD simulations: Interactions of Fmoc-FFpY/Fe^{3+} hydrogel with *S. aureus* membrane 10
9. Antibacterial activity of hydrogels and the peptide 15
10. Antibacterial activity of Fmoc-FFpY/Na+ hydrogels 16

1. Rheology

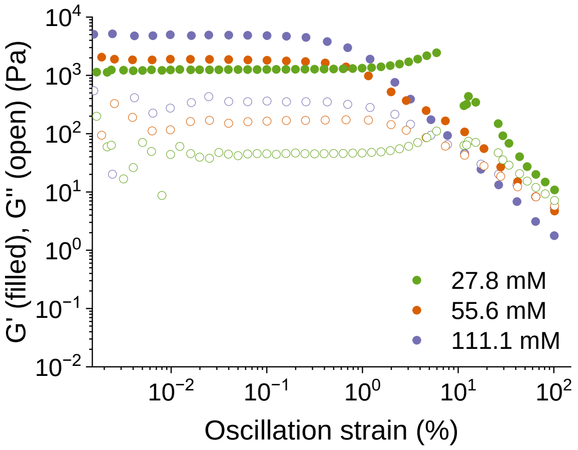

Figure S1. Rheological properties of Fmoc-FFpY/Fe^{3+} mixtures prepared at 5 mg/mL Fmoc-FFpY and different $FeCl_3$ concentrations: the storage modulus G' (filled symbols) and the loss modulus G'' (open symbols) as a function of the oscillation strain from 0.001% to 100% (1 Hz) at 25 °C.

2. Cryo-SEM images of Fmoc-FFpY/Fe^{3+} hydrogels

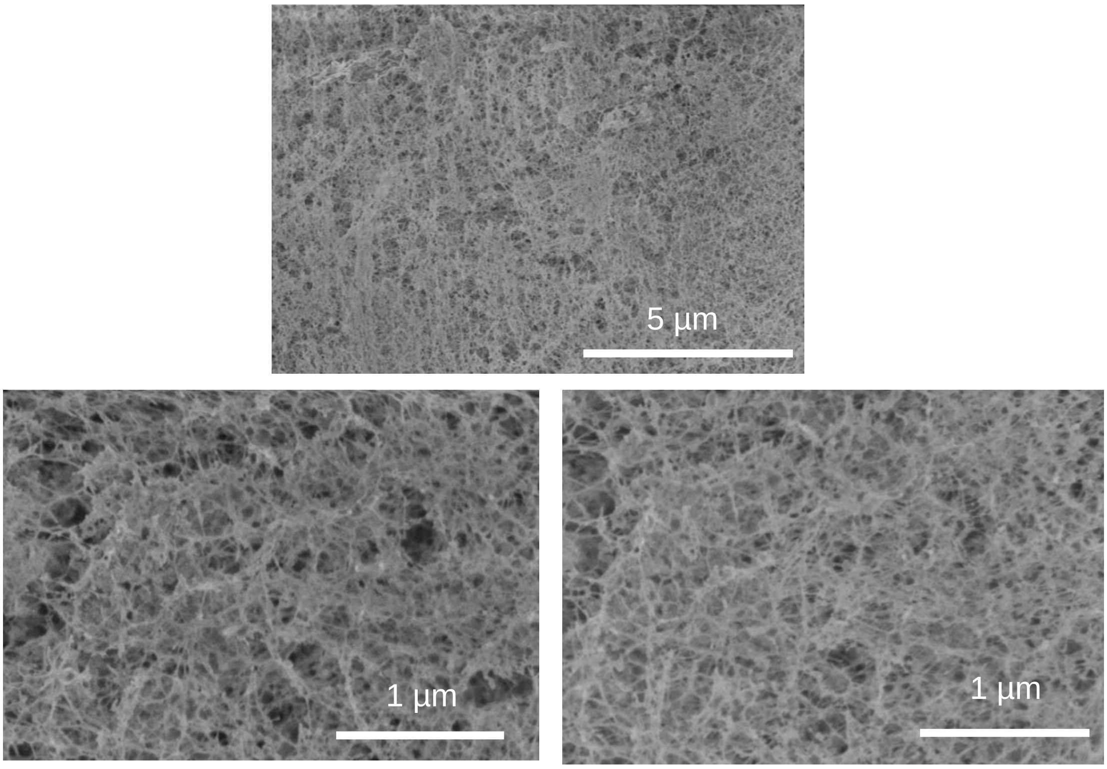

Figure S2: Morphology of Fmoc-FFpY/Fe^{3+} self-assembly: Cryo-SEM images of hydrogels prepared at 27.8 mM $FeCl_3$.

3. Molecular dynamics (MD) simulations: aggregation and supramolecular organization

Throughout the MD simulations, the energy, temperature, and volume parameters are monitored to ascertain if any technical issues arise during the simulation, a scenario that was not encountered during the calculations. Traditionally, in the MD of biological macromolecules, the root means square deviations (RMSD) along the trajectory are analyzed, serving to visualize the time required for the thermal equilibration of systems and evaluation of the global flexibility of the biological macromolecule. However, in cases where the observation of peptide aggregation is desired, this measure proves to be less informative as depicted on Figure S3a. In our scenario, a measurement of non-native contacts along the molecular dynamic's trajectory has been opted for. In MD, non-native contacts refer to interactions between atoms or residues within a biomolecular system that are not typically present in its native or biologically relevant structure. These contacts can arise due to various factors such as conformational fluctuations, transient interactions, deviations from the native state, or aggregation of peptides as in our case. The calculation of non-native contacts involves monitoring the distances between pairs of atoms or residues within the system throughout the simulation. If the distance between two atoms or residues falls below a certain threshold value, typically chosen based on the expected native contacts in the system (4 Å in our case), they are considered to be in contact. Figure S3b depicts the calculation made for a typical simulation.

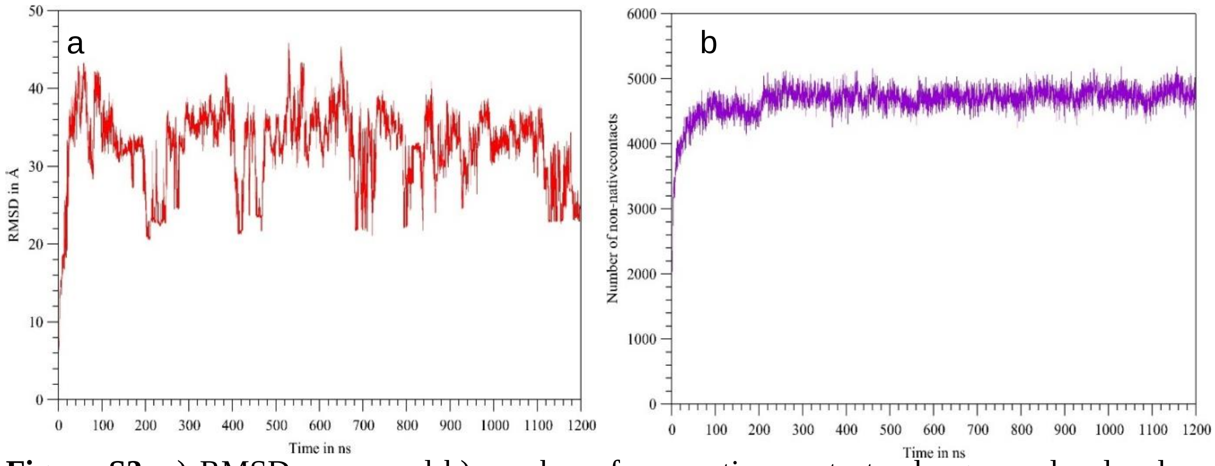

Figure S3. a) RMSD curve and b) number of non-native contacts along a molecular dynamics trajectory for a typical system.

In simulation, periodic conditions enable the consideration of environmental effects by replicating calculations across all directions. The calculation is performed within a simulation box at the center, which is then replicated across all three spatial directions (Figure S4a). In MD, where the movement of molecules is observed, it is common for a molecule to translate and end up in an image box. Subsequently, this molecule is reintroduced into the central simulation box through the same translation movement in the opposite image box. In most cases, these periodic conditions facilitate

the study of biological macromolecules (e.g. globular protein) in an aqueous environment, where periodicity ensures the maintenance of a constant solvent surrounding the protein.

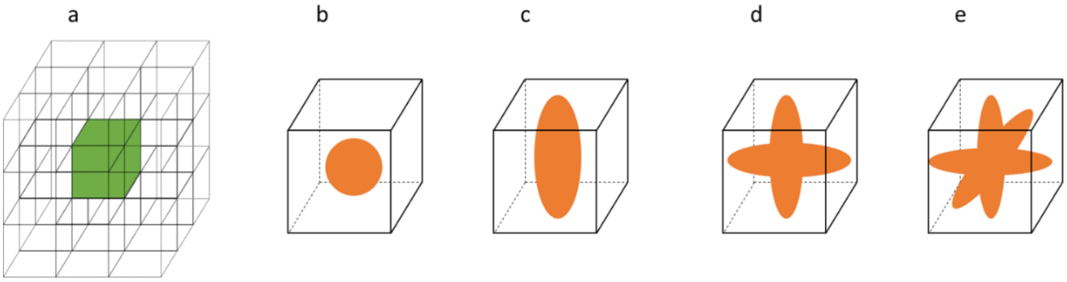

Figure S4: Periodic boundary conditions as used in MD simulations a), b), c), d), and e) refer to supramolecular organizations named, respectively, drop, filament, 2D, and 3D materials.

During the aggregation of Fmoc-FFpY peptides, our simulations capitalize on periodic conditions to organize themselves. This way, one can observe the formation of a drop (no interaction with periodic images, Figure S4b), a filament (interactions in only one direction, Figure S4c), a 2D material (Fmoc-FFpY interactions occur in two different axes, Figure S4d), and a 3D material (interactions in all periodic directions, Figure S4e). Table S1 compiles all simulations made for the system. It can be seen from Table S1 that there are several behaviors for each simulation. This reinforces our desire to reproduce the calculations on a large set of simulations to identify a clear statistical trend.

Table S1. Shapes determined from the 20 simulations of the system.

Number	Replica	Shape
**1**	1	filament
	2	2D
	3	weak filament
	4	2D
**2**	1	2D
	2	weak 2D
	3	2D
	4	filament
**3**	1	2D
	2	drop
	3	weak filament
	4	filament
**4**	1	weak filament
	2	weak 2D
	3	drop
	4	2D
**5**	1	weak filament
	2	filament
	3	2D
	4	filament

4. Infrared spectroscopy and MD simulation of the secondary structure

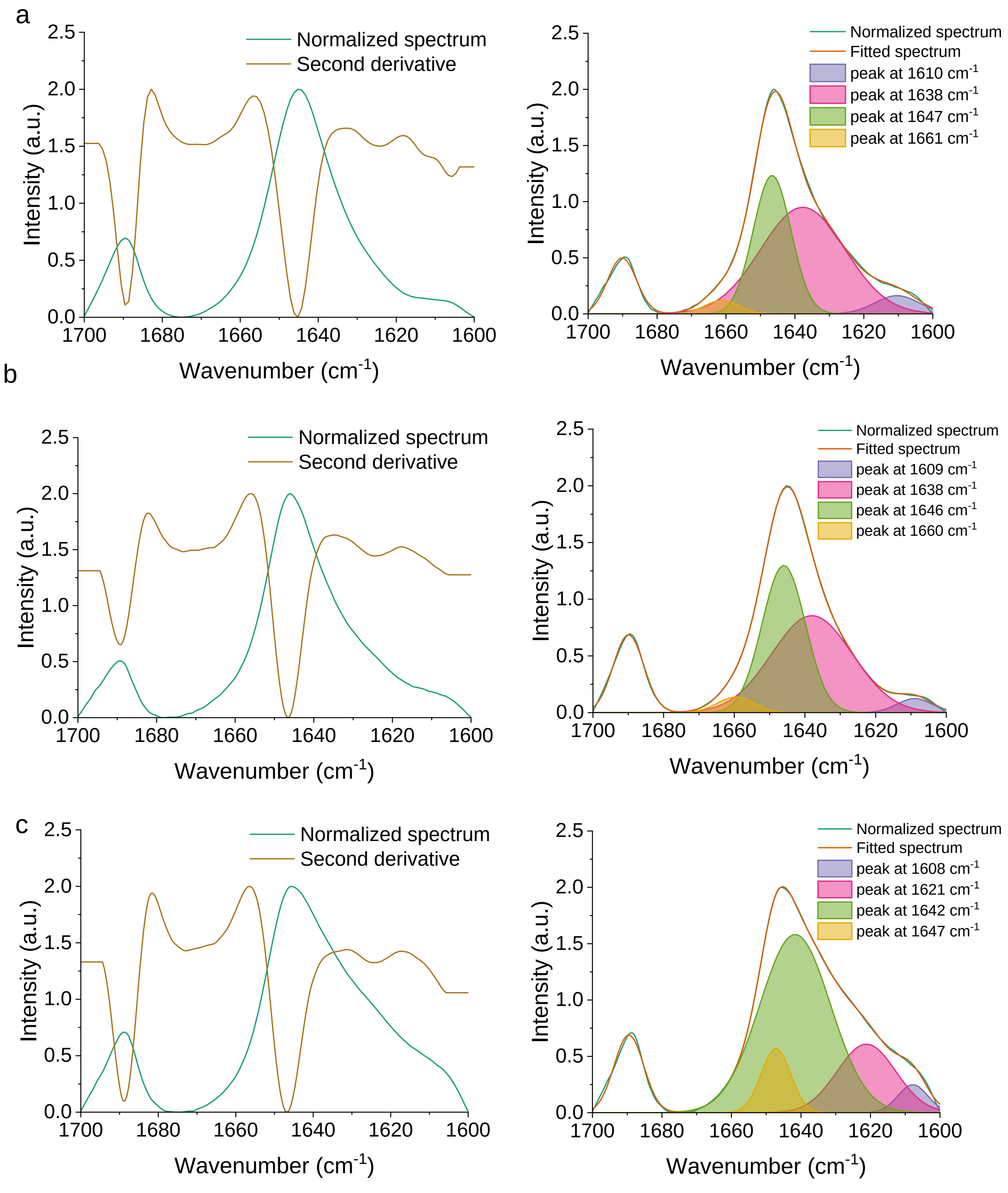

Figure S5. FTIR normalized spectra of Fmoc-FFpY/Fe^{3+} hydrogels, in dry state, prepared at a) 27.8 mM, b) 55.6 mM and c) 111.1 mM $FeCl_3$, with (left) the second derivative spectra obtained and (right) the fitting by multiple gaussian peaks using OPUS 7.5 software using the minimum positions of the second derivative.

Table S2. Relative content of the different secondary structure contributions of the amide I band of Fmoc-FFpY/Fe^{3+} hydrogels prepared at 27.8, 55.6 and 111.1 mM $FeCl_3$, determined by FTIR.

**[$FeCl_3$]**	**Structure**	**Peaks (cm^{-1})**	**Contribution in the amide I band (%)**
27.8 mM	β-sheet	1609	3
	β-sheet	1638	51
	Random coil	1646	42
	α-helix	1660	4
**[$FeCl_3$]**	**Structure**	**Peaks (cm^{-1})**	**Contribution in the amide I band (%)**
55.6 mM	β-sheet	1610	5
	β-sheet	1638	58
	Random coil	1647	34
	α-helix	1661	3
**[$FeCl_3$]**	**Structure**	**Peaks (cm^{-1})**	**Contribution in the amide I band (%)**
111.1 mM	β-sheet	1608	4
	β-sheet	1621	21
	Random coil	1642	65
	α-helix	1647	10

Ramachandran plots were employed to decipher the secondary structures of Fmoc-FFpY supramolecular organizations obtained from MD trajectories. This plot maps the dihedral angles (φ, ψ) of a peptide backbone and is a useful tool for determining the sterically allowed conformations of amino acid residues, thereby aiding in the prediction and validation of protein structures. Ramachandran plots were computed for the 20 MD simulations of the studied system and the secondary structure elements were ranged in α-helices, β-sheets, or coil conformations. Table S3 shows the percentage presence of the different supramolecular organizations of the Fmoc-FFpY peptides.

Table S3. Percentage of presence of the different secondary structures from the 20 simulations of the system.

Number	Replica	% of α-helix	% β-sheet	% random coil
1	1	2.5	57.5	40.0
	2	5.0	52.5	42.5
	3	7.5	57.5	35.0
	4	3.8	62.5	33.8
2	1	6.3	68.8	25.0
	2	7.5	70.0	22.5
	3	5.0	75.0	20.0
	4	3.8	73.8	22.5
3	1	7.5	47.5	45.0
	2	5.0	56.3	38.8
	3	1.3	61.3	37.5
	4	2.5	57.5	40.0
4	1	5.0	56.3	38.8
	2	6.3	52.5	41.3
	3	7.5	66.3	26.3
	4	3.8	62.5	33.8
5	1	5.0	60.0	35.0
	2	3.8	57.5	38.8
	3	7.5	58.8	33.8
	4	1.3	70.0	28.8
Average		4.9	61.2	34.0
Standard deviation		0.5	1.7	1.6

5. Circular dichroism

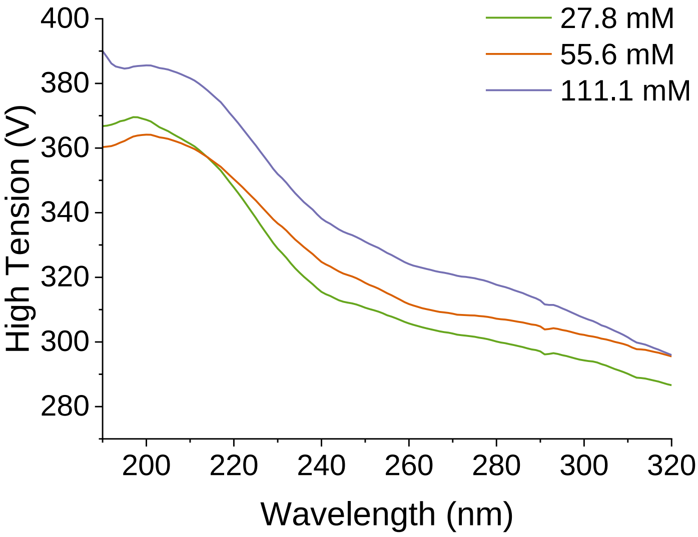

Figure S6. High tension as a function of the wavelength measured for Fmoc-FFpY/Fe^{3+} hydrogels at different $FeCl_3$ concentrations.

6. Molecular dynamics (MD) simulations: J-aggregates

In the review article by Hestand and Spano,[48] J-aggregates were defined based on two geometric criteria: (i) a distance of less than 5 Å between two aromatic rings and (ii) an angle of less than 54.7° ("magic angle") between the aromatic planes. To be considered a J-aggregate, it must persist for more than 20% of the total time during the simulation. The evaluation of J-aggregates was done over the 200 ns duration of each independent simulation.

Table S4. Number of J-aggregates for the simulation systems.

Number	Replica	Occurrence
1	1	25
	2	20
	3	23
	4	20
2	1	0
	2	27
	3	18
	4	20
3	1	6
	2	22
	3	4
	4	17
4	1	11
	2	5
	3	5
	4	11
5	1	19
	2	10
	3	27
	4	13
	average	15
	standard deviation	2

7. Stability of the Fmoc-FFpY/Fe^{3+} hydrogels in contact with PBS

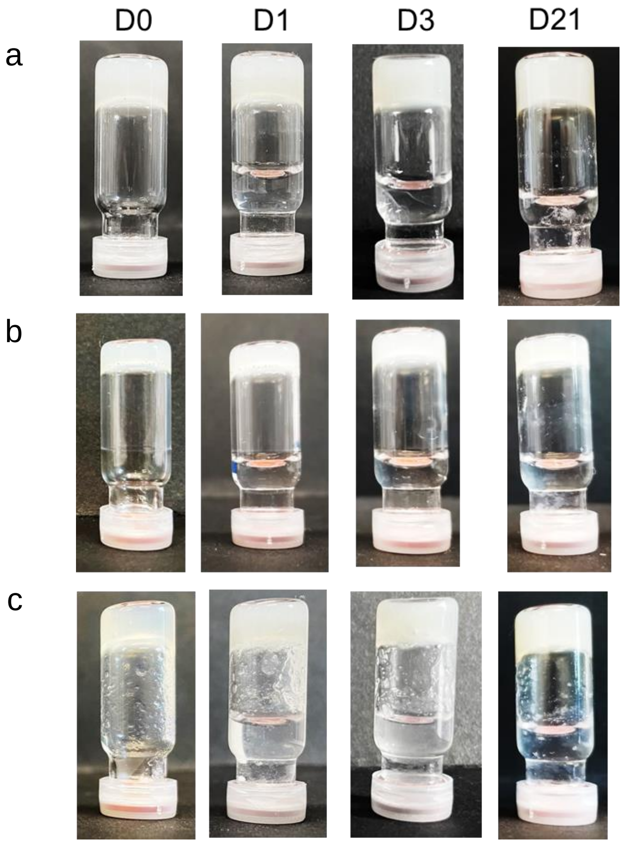

Figure S7. Inverted tube test of Fmoc-FFpY/Fe^{3+} hydrogels prepared at a) 27.8 mM, b) 55.6 mM, and c) 111.1 mM $FeCl_3$, before (D0) and after 27 days (D27) in contact with PBS at 37°C.

8. MD simulations: Interactions of Fmoc-FFpY/Fe^{3+} hydrogel with *S. aureus* membrane

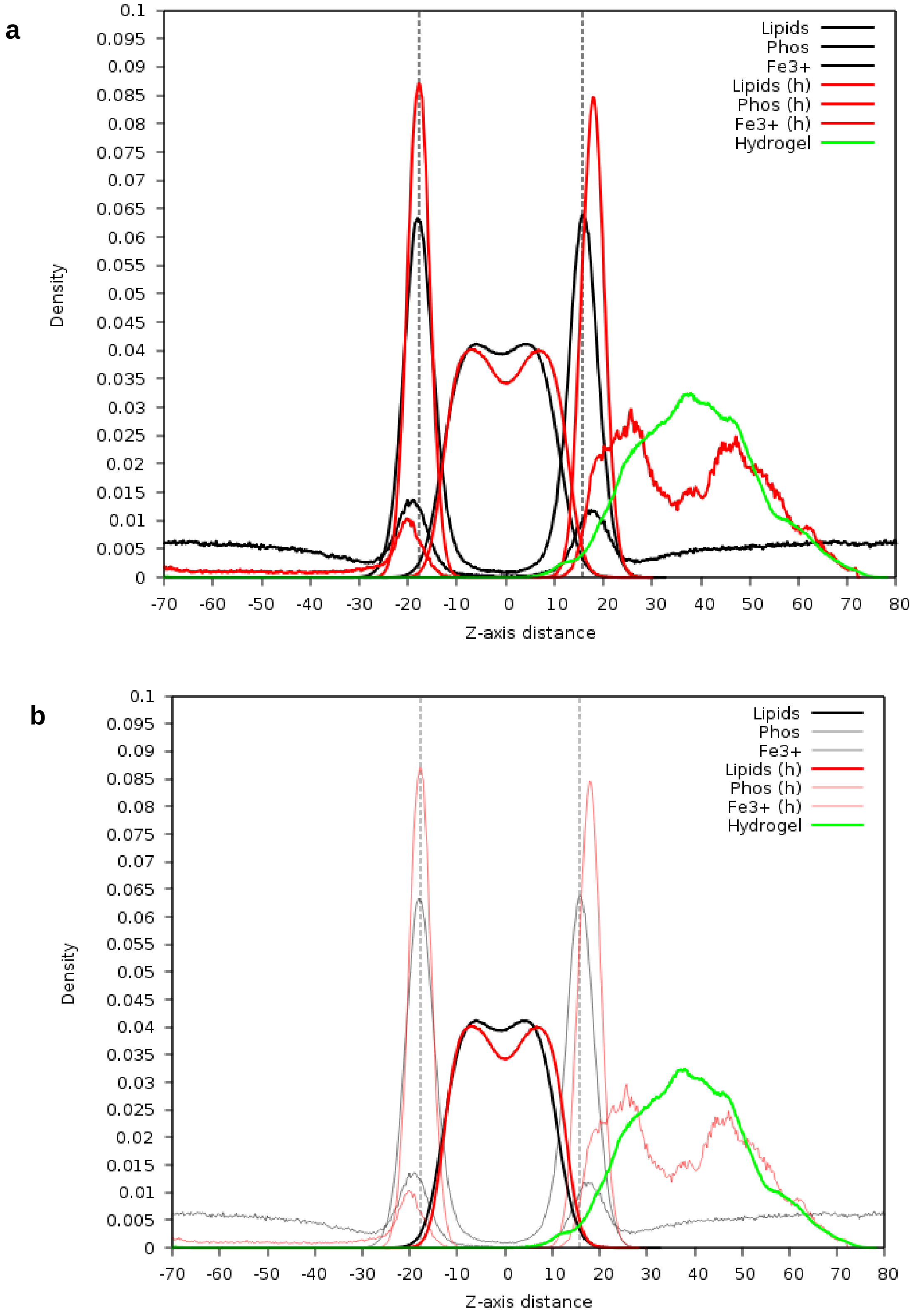

c

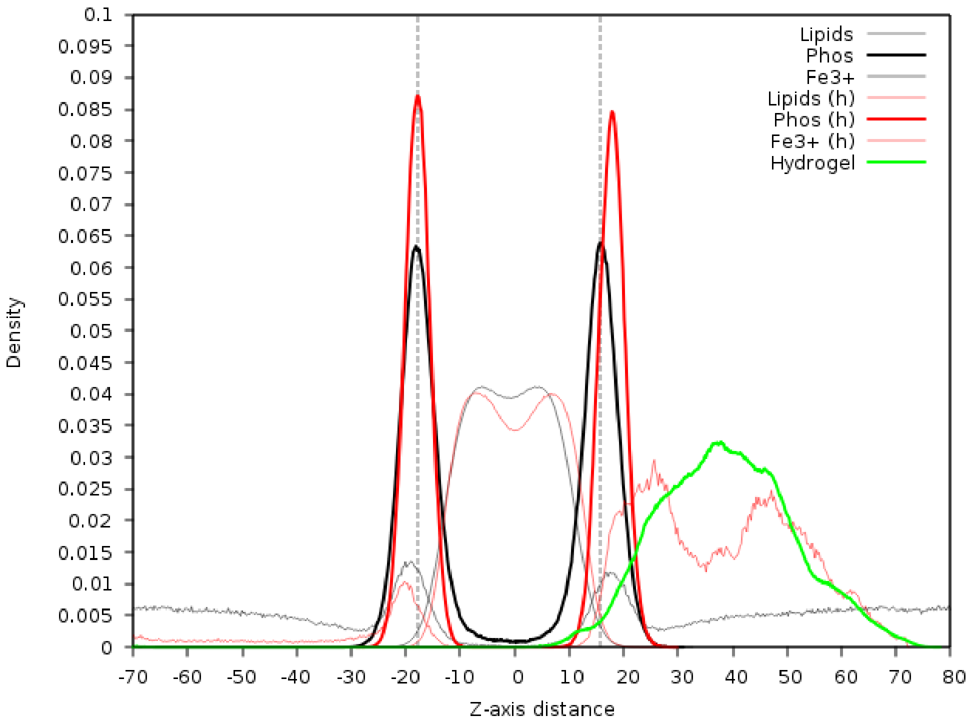

d

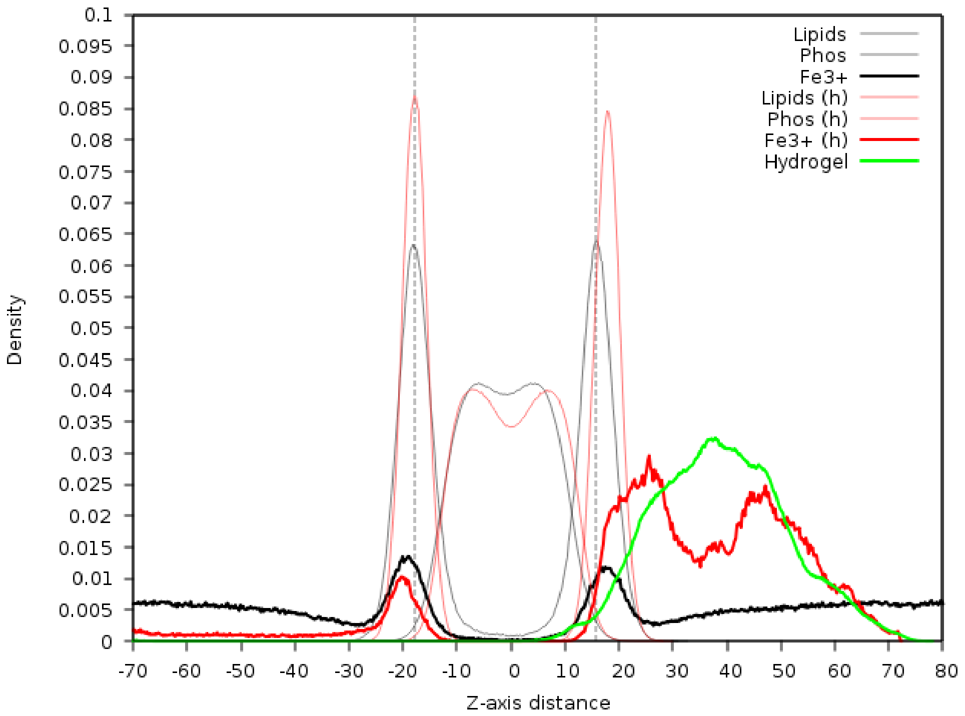

Figure S8: Density profile along the z-axis (in Å) determined and averaged from MD simulations of the *S. aureus* membrane in contact with $FeCl_3$ (in black) and with Fmoc-FFpY/$FeCl_3$ (in red, labelled (h)): a) the overall distributions, b) density of lipids, c) phosphorous atoms, and d) Fe^{3+} cations. The hydrogel density is shown in green.

a

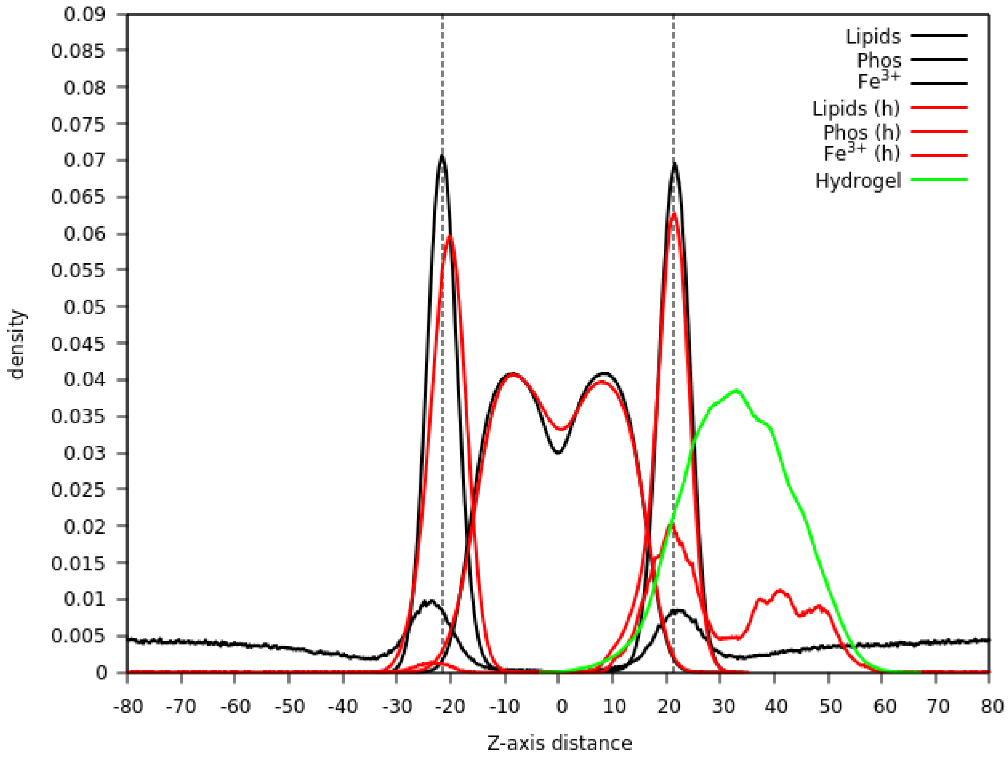
density
Z-axis distance
Lipids
Phos
Fe3+
Lipids (h)
Phos (h)
Fe3+ (h)
Hydrogel

b

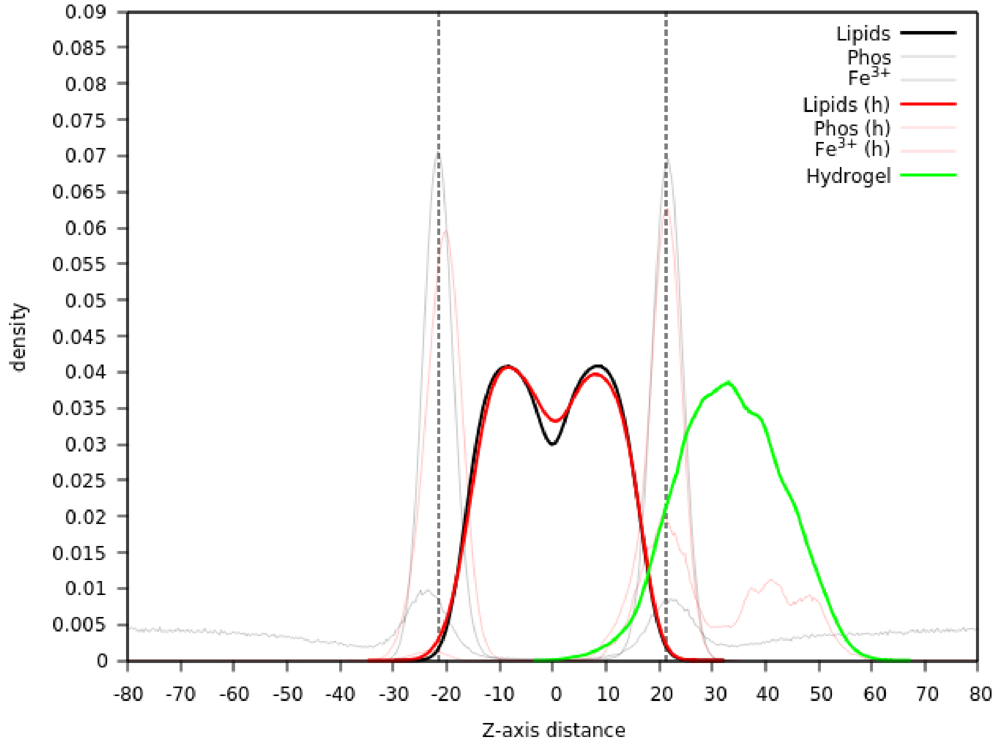
density
Z-axis distance
Lipids
Phos
Fe3+
Lipids (h)
Phos (h)
Fe3+ (h)
Hydrogel

c

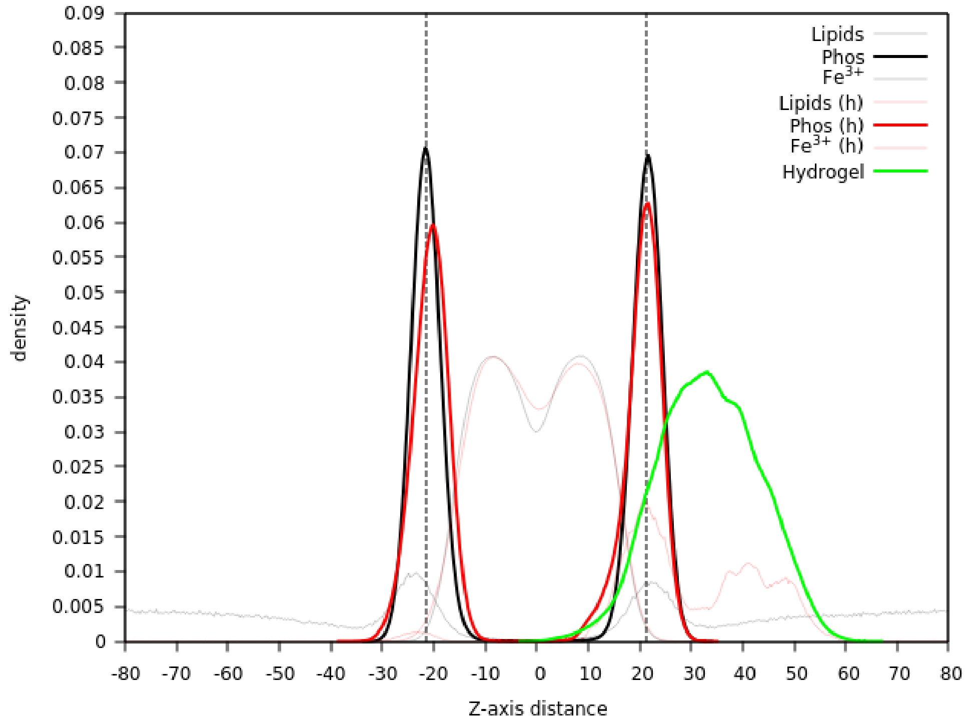

d

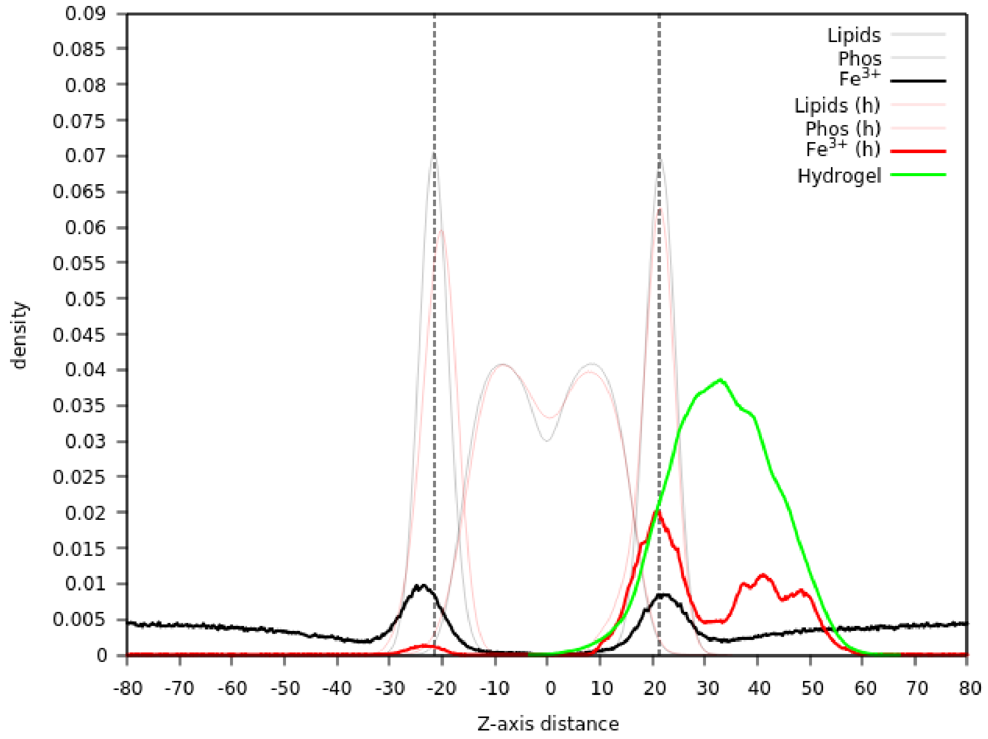

Figure S9: Density profile along the z-axis (in Å) determined and averaged from MD simulations of the *P. aeruginosa* membrane with $FeCl_3$ (in black) and Fmoc-FFpY/$FeCl_3$ (in red, labelled (h)): a) the overall distributions, b) density of lipids, c) phosphorous atoms, and d) Fe^{3+} cations. The hydrogel density is shown in green.

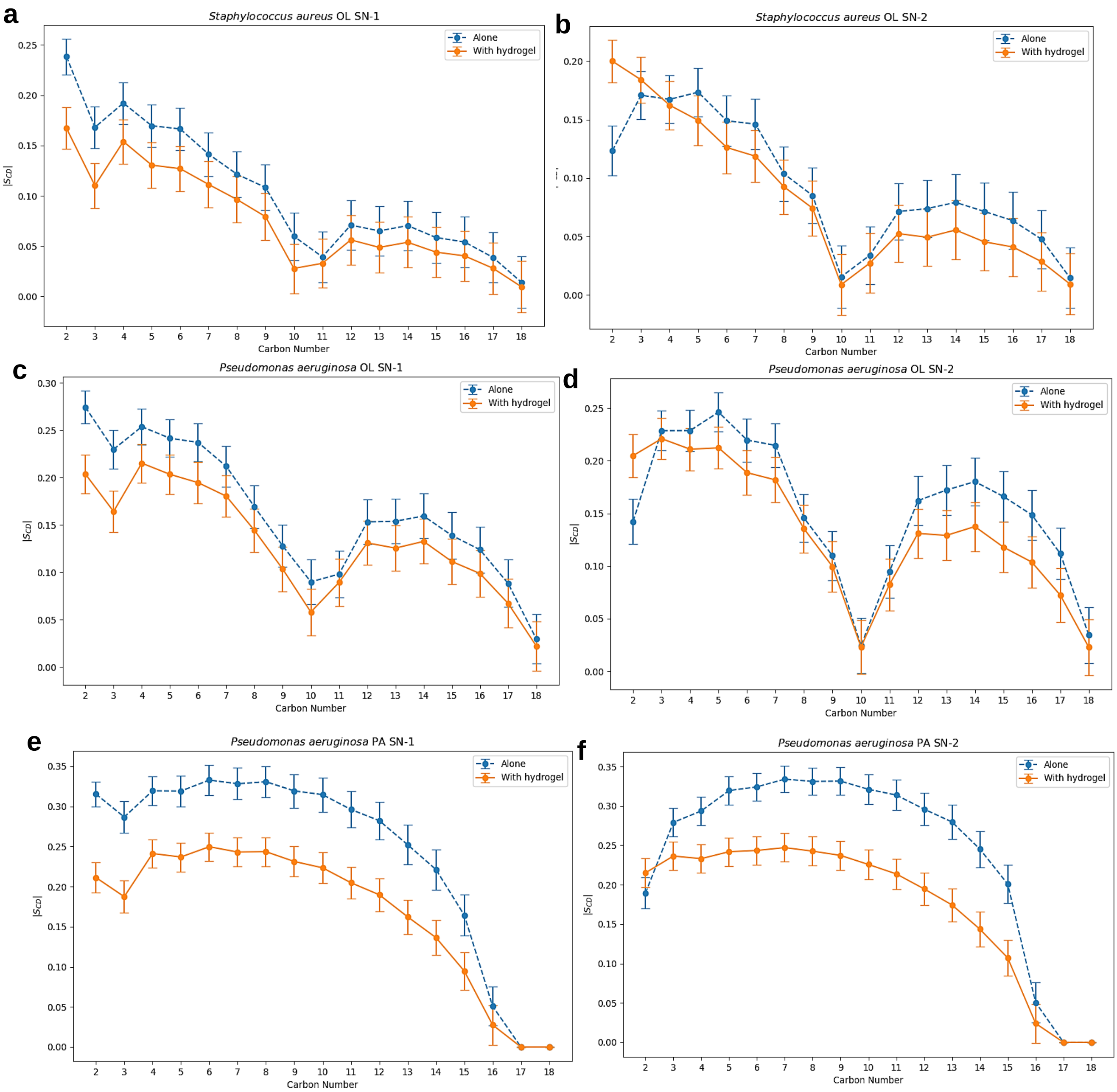

Figure S10: Deuterium order parameter (S_{CD}) of a-b) *S. aureus* and c-f) *P. aeruginosa* lipids bilayers of in the absence (blue dash line) and in the presence of Fmoc-FFpY/Fe^{3+} hydrogel (orange line). a) oleic lipid tail SN1 of *S. aureus* ; b) oleic lipid tail SN2 of *S. aureus* ; c) oleic lipid tail SN1 of *P. aeruginosa* ; d) oleic lipid tail SN2 of *P. aeruginosa* ; e) palmitic lipid tail SN1 of *P. aeruginosa* ; f) palmitic lipid tail SN2 of *P. aeruginosa*.

9. Antibacterial activity of hydrogels and the peptide

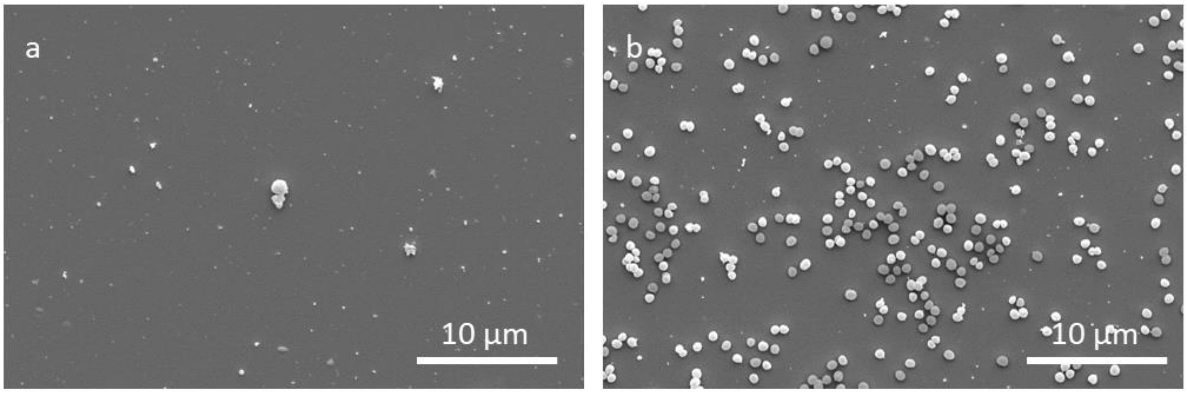

Figure S11: SEM images of *S. aureus* after 24 h contact with a) the hydrogel formulated at 111.1 mM $FeCl_3$ and b) 5 w/v% agar hydrogel.

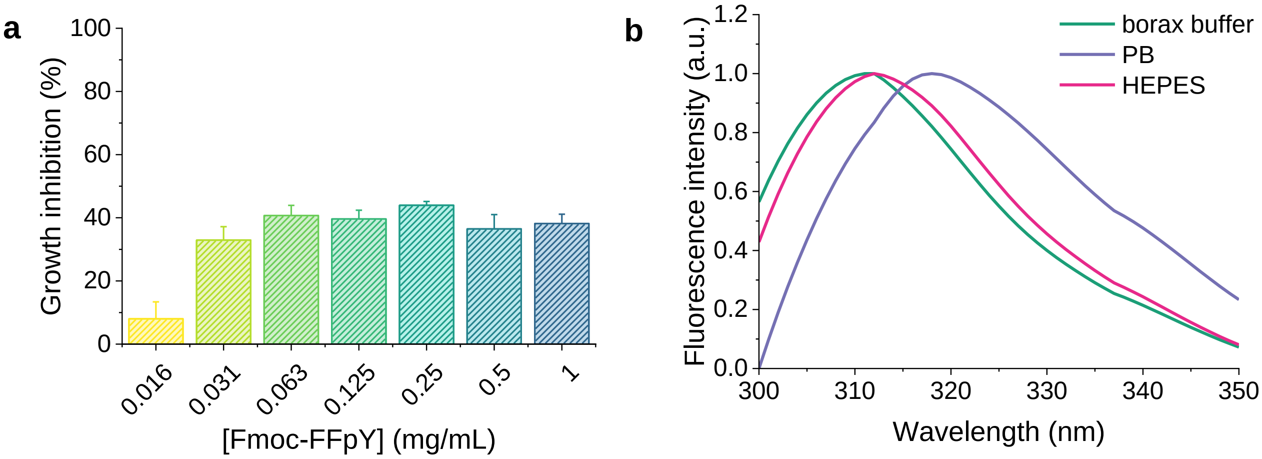

Figure S12. a) Growth inhibition of *S. aureus* in the presence of different concentrations of Fmoc-FFpY in MH broth medium. b) Normalized fluorescence emission spectra (λ_{EXC} = 290 nm) of Fmoc-FFpY (5 mg/mL) solution at pH 9.5 in 25 mM borax buffer and at pH 7.4 in 100 mM phosphate buffer (PB) and 1 M HEPES.

10. Antibacterial activity of Fmoc-FFpY/Na+ hydrogels

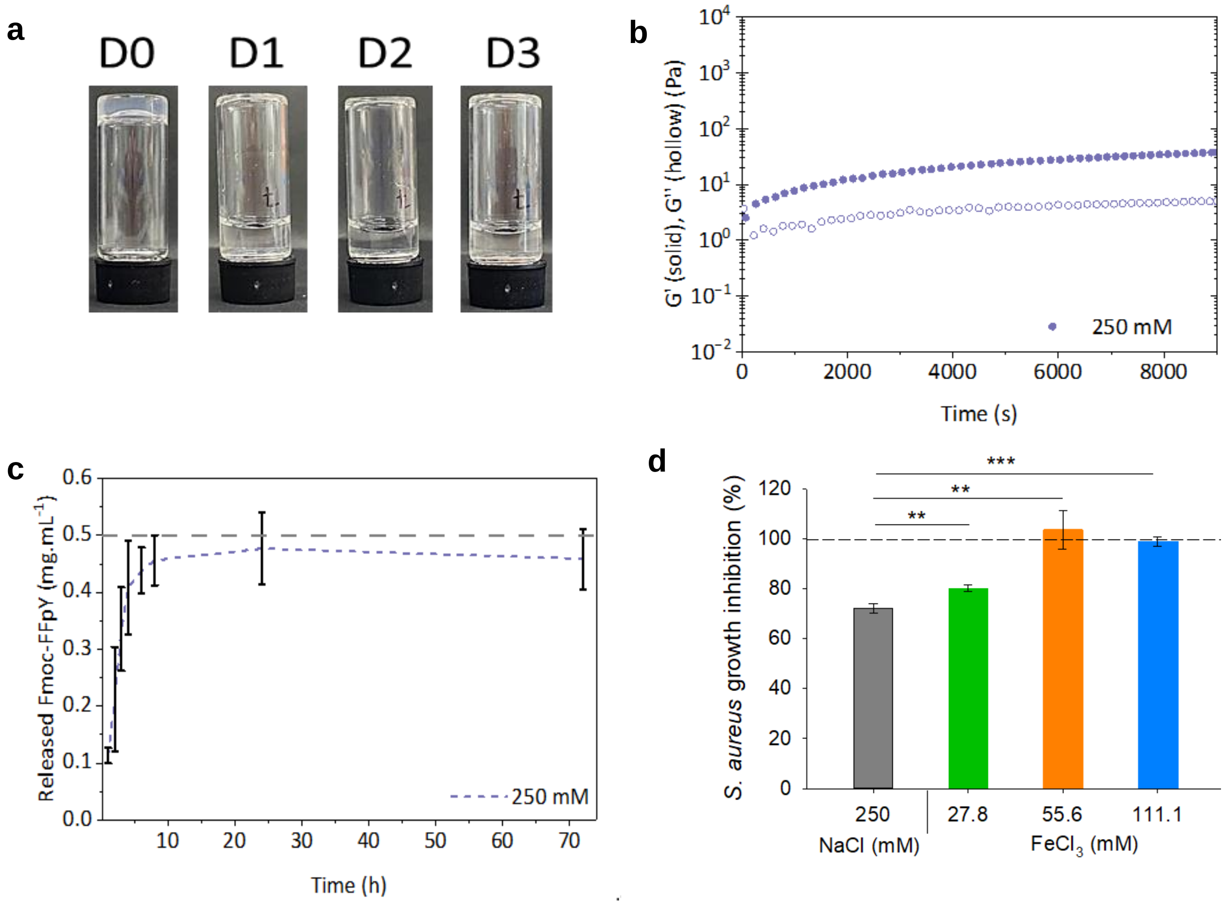

Figure S13: Fmoc-FFpY/Na^+ hydrogel, formulated at 250 mM NaCl. a) Inverted tube test before (D0) and after 1, 2 and 3 days (D1-3) of contact with PBS at 37°C b) rheological properties, storage modulus G' (filled symbols) and loss modulus G'' (open symbols), as a function of time obtained in a) time sweep (0.01% strain, 1 Hz) at 25 °C (inset: inverted tube test after 24 b) Cumulative amount of Fmoc-FFpY released in MH broth versus time (grey dashed line is the maximum peptide released in the case of a complete dissolution of the hydrogel), c) Growth inhibition of *S. aureus* in the presence of Fmoc-FFpY/Na^+ hydrogel compared to Fmoc-FFpY/Fe^{3+} hydrogels (n = 18). The dashed lines correspond to the positive control, *i.e.* agar gel and antibiotic mixture (tetracycline and cefotaxime). ** and *** symbols are for p-value of 0.01 and 0.001, respectively.
